# The Impact of Web-Based Ratings on Patient Choice of a Primary Care Physician Versus a Specialist: Randomized Controlled Experiment

**DOI:** 10.2196/11188

**Published:** 2019-06-28

**Authors:** Siyue Li, Austin Hubner

**Affiliations:** 1 College of Media and International Culture Zhejiang University Hangzhou China; 2 School of Communication The Ohio State University Columbus, OH United States

**Keywords:** technical skills, interpersonal skills, physician ratings, physician selection

## Abstract

**Background:**

Physician review websites have empowered prospective patients to acquire information about physicians. However, little is known about how Web-based ratings on different aspects of a physician may affect patients’ selection of physicians differently.

**Objective:**

The objectives of this study were to examine (1) how patients weigh ratings on a physician’s technical skills and interpersonal skills in their selection of physicians and (2) whether and how people’s choice of a primary care physician versus a specialist is affected differently by Web-based ratings.

**Methods:**

A 2×2×2×2 between-subjects experiment was conducted. Over 600 participants were recruited through a crowdsourcing website and randomly assigned to view a mockup physician review Web page that contained information on a physician’s basic information and patients’ ratings. After reviewing the Web page, participants were asked to complete a survey on their perceptions of the physician and willingness to seek health care from the physician.

**Results:**

The results showed that participants were more willing to choose a physician with higher ratings on technical skills than on interpersonal skills compared with a physician with higher ratings on interpersonal skills than on technical skills, *t*_369.96_=22.36, *P*<.001, Cohen *d*=1.22. In the selection of different types of physicians, patients were more likely to choose a specialist with higher ratings on technical skills than on interpersonal skills, compared with a primary care physician with the same ratings, *F*_1,521_=5.34, *P*=.021.

**Conclusions:**

The findings suggest that people place more weight on technical skills than interpersonal skills in their selection of a physician based on their ratings on the Web. Specifically, people are more likely to make a compromise on interpersonal skills in their choice of a specialist compared with a primary care physician. This study emphasizes the importance of examining Web-based physician ratings in a more nuanced way in relation to the selection of different types of physicians.

**Trial Registration:**

ISRCTN Registry ISRCTN91316463; http://www.isrctn.com/ISRCTN91316463

## Introduction

### Background

The role of patients in health care is undergoing a remarkable transition. Although traditional patients took a passive role in their health care, modern patients are actively involved in health decision making [1,2]. For instance, patients are increasingly turning to Web-based physician review websites (PRWs) to learn information about their physicians [3,4]. Indeed, a national survey suggests that more than half of the respondents consider PRWs as an important source for information when choosing a physician [3]. These websites not only provide information about a physician’s basic information and qualifications but also present peer-to-peer consumer reviews of the physician. On the basis of the reviews, health consumers are able to learn about other patients’ experiences, concerns, and levels of satisfaction about a specific physician.

With the growing popularity of PRWs, researchers have recently begun to examine the role of these websites in people’s health decision making [5-8]. The scholarship on PRWs covers a wide range of topics, including but not limited to demographics of website users, structures of the portals, patterns of website usage, and content of reviews [9-11]. Technical skills and interpersonal skills reside at the core of a physician’s qualifications and are commonly rated on PRWs [12,13]. However, little is known about how ratings on these different aspects of a physician may affect patients’ choice differently [14]. Previous research presents mixed results on how people set the priority of technical and interpersonal skills in physician selection [15,16]. Therefore, the first goal of this study was to examine how patients prioritize technical and interpersonal skills in their physician selection based on ratings on PRWs.

The second objective of this study was to examine whether and how people’s choice of a primary care physician versus a specialist is affected differently by Web-based ratings. In the United States, patients are allowed greater autonomy to choose their primary care physicians compared with specialists. As a result, significantly more research focuses on people’s selection of primary care physicians than physicians of other types [16-18]. Nowadays, however, patients are more involved in the choice of specialists in part because of the easy access of health information on the Web. Given that primary care physicians and specialists take on different roles in health care, patients may apply different criteria to select different types of physicians [3]. In this study, we specifically examine how ratings on a physician’s technical and interpersonal skills may affect patients’ choice of primary care physicians and specialists differently. We only examine medical doctors and exclude dentists because medicine and dentistry are often considered separately in health care. They involve different education systems, physician networks, medical records, and payment systems. Some PRWs list dentist as a different category from medical doctors and other sites might not include dentists.

### Technical Versus Interpersonal Skills

In the era of health consumerism, people tend to evaluate a wide array of factors in their selection of physicians. Research on physician selection criteria has shown that people not only consider the factors pertaining to a physician (eg, sex, age, race, and qualification) but also evaluate many other contextual and economic factors, such as office location and insurance coverage [12,16,18].

Despite a large collection of factors to consider, technical skills and interpersonal skills are central to the evaluation of a physician’s qualifications [12,13]. Technical skills concern medical knowledge and expertise in a physician’s area. Overall, patients prefer a physician who is skilled and knowledgeable in their domain of expertise with the ability to provide accurate diagnosis and treatment [12]. Interpersonal skills center on the communication style of a physician [19]. Especially with the recent push for patient-centered care, physicians of all types are reevaluating their approach to communicating with patients [20,21]. Patients, in general, prefer a physician who is easy to talk to and willing to listen [22]. Physicians with a caring and friendly style lead to high levels of patient satisfaction [23].

Although both technical and interpersonal skills are important considerations in patients’ choice of physicians, it is unclear how patients set the priority of the 2 factors. Research has presented inconsistent findings regarding patients’ selection criteria of a physician [15,16,24,25]. A body of literature focusing on primary care physicians found that people expressed a clear preference for technical skills over interpersonal skills [12,26]. However, other studies show that communication skills are the most important determinant in patients’ choice of a primary care physician [15,16,22]. Various aspects of interpersonal skills, such as a caring attitude and responsiveness, are found to be preferred over technical skills in people’s choice of a primary care physician [16,27].

Similarly, research on patients’ selection of specialists has generated mixed findings on patients’ preferences on technical skills versus interpersonal skills [13,24,25]. Hoerger and Howard [25] found that women rated medical expertise as the leading reason for choice of a prenatal care physician. On the contrary, Dunlea and Lenert [24] surveyed people over their preference of a specialist with a hypothetical referral of an asymptomatic condition and concluded that communication skills and the shared decision-making style were considered more important than medical expertise in their choice.

PRWs provide prospective patients with valuable information regarding physicians. The valence of Web-based reviews tends to affect people’s perceptions of physicians and their intention to choose the physicians [10]. The decision is even more complicated when people are exposed to reviews with opposite valence in different skills. For example, physicians may receive positive feedback on their technical competence but negative or neutral reviews on their interpersonal skills (or vice versa). Under such circumstances, patients may need to make tradeoffs between a physician’s technical and interpersonal skills. As previous research presented conflicting findings on patients’ preference over a physician’s technical skills versus interpersonal skills, the following research question was raised:

RQ1: Are people more willing to choose a physician with higher ratings on technical skills than on interpersonal skills, or a physician with higher ratings on interpersonal skills than on technical skills?

### Physician Types and Skills

Extant literature has demonstrated the importance of technical skills and interpersonal skills in people’s consideration of a physician, regardless of specialty [13]. However, the relative importance of technical and interpersonal skills may vary as a function of physician types. First, primary care physicians and specialists provide distinct medical services that require different levels of technical skills. In the United States, patients visit primary care physicians mainly for comprehensive care at the point of first contact, whereas they count on specialists for more specialized and advanced care. Primary care physicians generally have a wide range of medical knowledge and offer comprehensive care to patients, but they may not have advanced training for a particular domain of health. Specialists, as compared with primary care physicians, have advanced training in a particular branch of medicine (eg, bone or lung health) and are capable of providing more specialized care for patients. Patients who need advanced care for specific health conditions are usually referred by their primary care physicians to a specialist. Owing to the distinct services provided by primary care physicians and specialists, patients’ expectations for the technical skills of their physicians may vary between the types. Technical skills are likely more important in the assessment of specialists compared with primary care physicians.

Beyond differing responsibilities in health care, primary care physicians and specialists differ in their relationships with patients. Long-term relationships are typically expected with primary care physicians whereas specialist-patient relationships are largely bound by specific health problems and in many cases are short-lived [13,28]. Many patients choose to stay with the same primary care physician for an extended period of time. These relationships, for instance, might last over 10 years [29]. On the contrary, patients who are having a specific health condition treated may not need to keep a long-term relationship with their specialists. As sophisticated interpersonal skills are found to be important factors for building long-term relationships [30], patients may place more weight on interpersonal skills in their selection of a primary care physician compared with a specialist.

A limited body of literature has compared the relative importance of technical and interpersonal skills in people’s choice of primary care physicians and specialists. Hanna et al [31] found that communication skills were the leading factor in the selection of a primary care physician, whereas medical expertise was the key determinant in the choice of a specialist. In addition, a study on female patients’ selection of a primary care physician versus a specialist showed that people rated medical expertise to be a more important determinant in selection of a surgeon compared with a primary care physician [13]. Although we know little about the comparison between primary care physicians and specialists based on their skills, the sparse research evidence indicates that technical skills are likely to be more valued in the selection of specialists and interpersonal skills to be more valued in the selection of primary care physicians. When physicians receive Web-based ratings that indicate different qualities of their technical and interpersonal skills, patients may need to make a tradeoff. It is expected that patients are more willing to sacrifice interpersonal skills for technical skills in their selection of a specialist compared with a primary care physician. In contrast, patients are more likely to make a compromise on technical skills than interpersonal skills in their selection of a primary care physician versus a specialist. The following hypotheses were proposed:

H1: People are more willing to choose a specialist who has higher ratings on technical skills than on interpersonal skills, compared with a primary care physician who has the same ratings.

H2: People are more willing to choose a primary care physician who has higher ratings on interpersonal skills than on technical skills, compared with a specialist who has the same ratings.

## Methods

### Ethical Approval

The Institutional Review Board at the Ohio State University approved all study procedures.

### Sample

A total of 608 participants completed the Web-based experiment. Participants were recruited via the crowdsourcing website, Amazon’s Mechanical Turk (mTurk), and compensated for their time. We excluded people who failed the attention checks (n=26) and those who spent no time (n=1) or less than 5 seconds on the Web page (n=41). Of the 540 valid cases, 300 (300/540, 55.6%) were male and 239 (239/540, 44.3%) were female, with an average age of 35.83 (SD 11.30) years. The majority of the participants indicated that they were Caucasian (402/40, 74.4%), followed by Asian/Asian American (60/540, 11.1%), Hispanic/Latino (32/540, 5.9%), and African American (31/540, 5.7%). Demographics are provided in Table 1.

**Table:** 

Characteristics		Total	Experimental group							
			Primary care physician				Specialist			
			Moderate ratings on technical skills		High ratings on technical skills		Moderate ratings on technical skills		High ratings on technical skills	
			IS^a^, Moderate (n=65)	IS, High (n=69)	IS, Moderate (n=70)	IS, High (n=69)	IS, Moderate (n=69)	IS, High (n=67)	IS, Moderate (n=67)	IS, High (n=64)
**Gender, n (%)**										
	Male	300 (55.6)	39 (60)	33 (47.8)	39 (55.7)	39 (56.5)	37 (53.6)	32 (47.8)	43 (64.2)	38 (59.4)
	Female	239 (44.3)	26 (40)	36 (52.2)	31 (44.3)	30 (43.5)	31 (44.9)	35 (52.2)	24 (35.8)	26 (40.6)
	Unspecified	1 (0.2)	0 (0)	0 (0)	0 (0)	0 (0)	1 (1.4)	0 (0)	0 (0)	0 (0)
**Education, n (%)**										
	High school graduate or less	74 (13.7)	9 (13.8)	6 (8.7)	12 (17.1)	8 (11.6)	10 (14.5)	10 (14.9)	7 (10.4)	12 (18.8)
	Some college	135 (25)	12 (18.5)	25 (36.2)	20 (28.6)	15 (21.7)	14 (20.3)	17 (25.4)	18 (26.9)	14 (21.9)
	2-years degree	84 (15.6)	13 (20)	5 (7.2)	11 (15.7)	11 (15.9)	12 (17.4)	9 (13.4)	12 (17.9)	11 (17.2)
	Bachelor’s degree	190 (35.2)	23 (35.4)	27 (39.1)	18 (25.7)	23 (33.3)	26 (37.7)	27 (40.3)	22 (32.8)	24 (37.5)
	Graduate degree	57 (10.6)	8 (12.3)	6 (8.6)	9 (12.8)	12 (17.4)	7 (10.1)	4 (6.0)	8 (11.9)	3 (4.7)
Age (years), mean (SD)		35.8 (11.3)	35 (10.6)	34.6 (12.5)	36.5 (10.1)	37.2 (13.1)	33.8 (9.2)	36.4 (10.8)	37.1 (12.3)	36.0 (11.2)
**Income, n (%)**										
	Less than 20,000	100 (18.5)	11 (16.9)	12 (17.4)	16 (22.9)	14 (20.3)	16 (23.2)	13 (19.4)	8 (11.9)	10 (15.6)
	20,000 to <40,000	163 (30.2)	17 (26.1)	22 (31.9)	20 (28.6)	23 (33.3)	24 (34.8)	16 (23.9)	21 (31.3)	20 (31.2)
	40,000 to <60,000	114 (21.1)	17 (26.1)	14 (20.3)	10 (14.3)	16 (23.2)	15 (21.7)	13 (19.4)	11 (16.4)	18 (28.1)
	60,000 to <80,000	80 (14.8)	6 (9.2)	10 (14.4)	14 (20.0)	6 (8.7)	9 (13)	13 (19.4)	13 (19.4)	9 (14.1)
	80,000 to <100,000	39 (7.2)	5 (7.7)	3 (4.3)	8 (11.4)	6 (8.7)	1 (1.4)	6 (9.0)	6 (9.0)	4 (6.3)
	100,000 and higher	44 (8.1)	9 (13.8)	8 (11.6)	2 (2.9)	4 (5.8)	4 (5.8)	6 (9.0)	8 (11.9)	3 (4.7)
**Race, n (%)**										
	Caucasian	402 (74.4)	52 (80)	51 (73.9)	52 (74.3)	46 (66.7)	55 (79.7)	50 (74.6)	50 (74.6)	46 (71.9)
	Hispanic	32 (5.9)	1 (1.5)	2 (2.9)	4 (5.7)	4 (5.8)	3 (4.3)	5 (7.5)	4 (6.0)	9 (14.1)
	African American	31 (5.7)	6 (9.2)	6 (8.7)	4 (5.7)	4 (5.8)	4 (5.8)	2 (3.0)	2 (3.0)	3 (4.7)
	Native American/ Alaskan Native	5 (0.9)	0 (0)	0 (0)	0 (0)	1 (1.4)	2 (2.9)	0 (0)	2 (3.0)	0 (0)
	Asian	60 (11.1)	5 (7.7)	9 (13)	7 (10)	14 (20.3)	3 (4.3)	9 (13.4)	8 (11.9)	5 (7.8)
	Middle Eastern	2 (0.4)	1 (1.5)	0 (0)	1 (1.4)	0 (0)	0 (0)	0 (0)	0 (0)	0 (0)
	Pacific Islander	1 (0.2)	0 (0)	0 (0)	0 (0)	0 (0)	0 (0)	0 (0)	1 (1.5)	0 (0)
	Other	6 (1.1)	0 (0)	1 (1.4)	2 (2.9)	0 (0)	1 (1.4)	1 (1.5)	0 (0)	1 (1.6)

^a^IS: interpersonal skills

### Research Design

To investigate the proposed research question and hypotheses, a 2 (ratings on interpersonal skills: high versus moderate) × 2 (ratings on technical skills: high versus moderate) × 2 (physician specialty: primary care physician versus specialist) × 2 (order of ratings: interpersonal skills first versus technical skills first) between-subjects factorial design was employed. Participants were randomly assigned to one of the 16 experimental conditions and instructed to read through a cover story describing a medical condition in which they need to find a new physician. They were then asked to view a mockup physician review page and complete a questionnaire about their perceptions of the reviewed physician and their willingness to choose the physician.

### Stimulus Materials

Following consent, participants were presented a cover story to read. On the basis of the type of physician that they were assigned to, participants were asked to imagine themselves in a situation looking for either a primary care physician or a surgeon. The vignette about a primary care physician depicted a situation that the participant recently moved to a new city and was in need of a new primary care physician. Owing to a lack of input from family members and friends, they decided to search for primary care physicians on PRWs. The vignette about a surgeon described a situation in which the participant had lasting back pains. The primary care physician suspected that the patient may need spinal surgery and provided a list of surgeons to choose from. The participant decided to search for the recommended surgeons on PRWs. After reading through the scenario and imaging themselves in the described situation, each participant was directed to a physician review page to learn about the physician.

**Figure 1:**
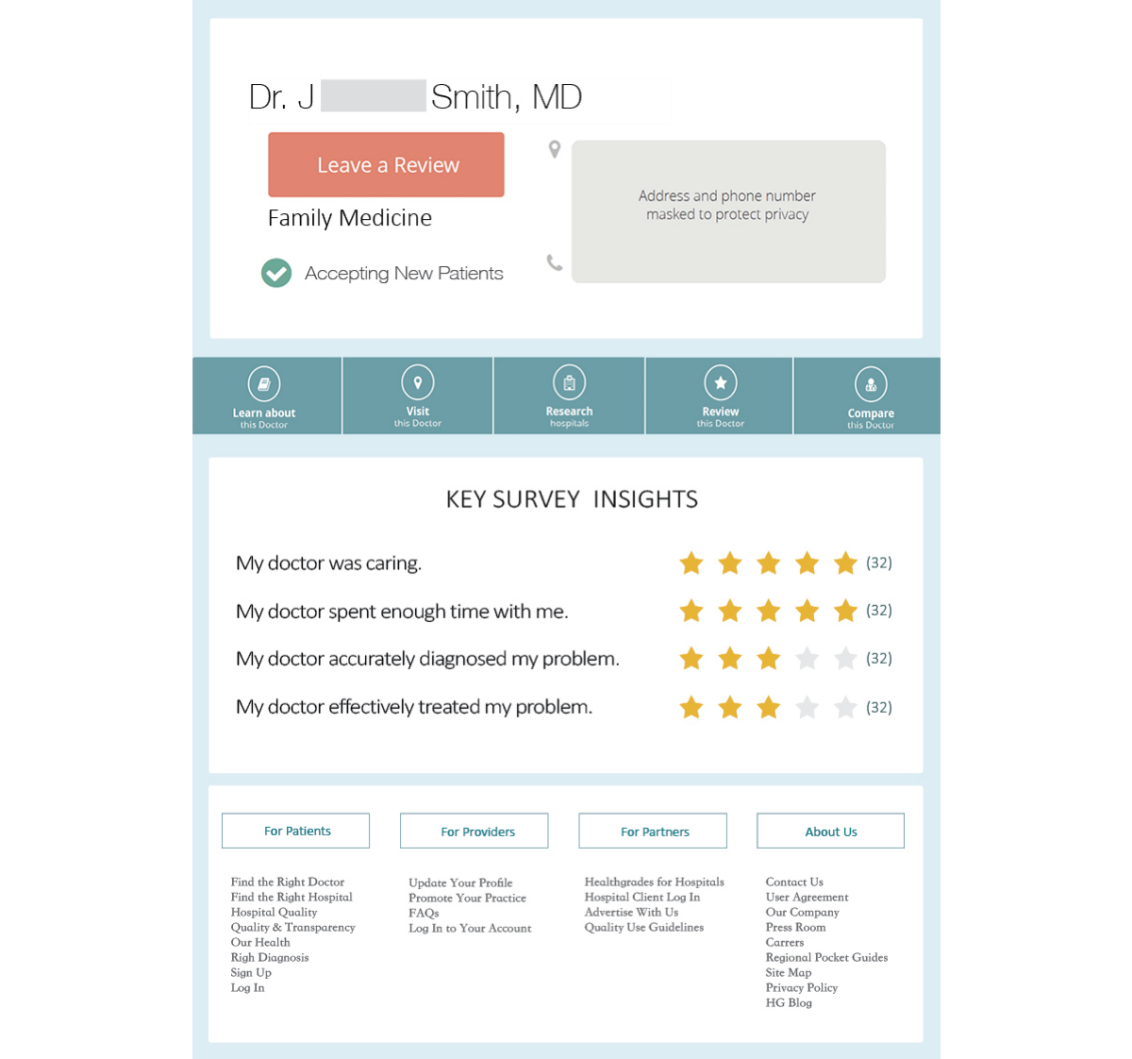
An example of the physician review page stimuli.

A total of 16 physician review pages were developed for this study (see Figure 1). The top part of each page listed basic information about a physician, including the physician’s name (Dr J Smith), the specialty (family medicine or surgeon), and information on new patient acceptance (accepting new patients). To manipulate the type of a physician, half of the Web pages listed the physician’s specialty as family medicine and the other half described the physician as a surgeon.

Each page contained 4 aggregated rating categories about Dr Smith, including 2 items on technical skills (“My doctor accurately diagnosed my problem” and “My doctor effectively treated my problem”) and 2 on interpersonal skills (“My doctor was caring” and “My doctor spent enough time with me”). Past research has suggested that a physician’s skills on diagnosis and treatment are among the most important considerations when selecting a physician [32]. In addition, a physician’s personal manner as well as time spent with a patient are critical to a patient’s satisfaction on the physician’s interpersonal skills [32,33]. These 4 categories frequently appear on PRWs [34] and thus are adopted in this study. To manipulate the valence of physician ratings, these rating categories were assigned different star ratings. Each rating category was presented in the form of aggregated ratings. In the conditions where a physician received high ratings on technical skills, the 2 items pertaining to technical skills were given 5/5-star ratings. In the conditions of moderate ratings on technical skills, the same items were assigned 3/5-star ratings. We chose to examine moderate ratings instead of low ratings in this study because research suggests that low ratings are relatively uncommon on PRWs . The valence of a physician’s interpersonal skills was manipulated in the same way. Furthermore, the rating categories were presented to participants in counterbalanced order to control for the impact of rating order effects. In half of the experimental conditions, the 2 rating categories on technical skills were displayed before the 2 categories on interpersonal skills. In the other half, ratings on technical skills were presented beneath the ratings on interpersonal skills.

### Measures

#### Willingness to Choose a Physician

A participant’s intention to choose the reviewed physician was assessed with 3 items on a 7-point scale (1=would definitely not choose/definitely unwilling; 7=would definitely choose/definitely willing). The 3 items are “How likely is it that you would choose someone like Dr Smith to be your primary care doctor or surgeon?” (depending on the assigned physician condition), “How willing would you be to go to a doctor like Dr Smith for your medical care?”, and “How willing would you be to recommend a doctor like Dr Smith to your family member and friends if they have the need?” An exploratory factor analysis yielded only 1 factor with an eigenvalue greater than 1, explaining 94.17% of the total variance. All 3 items have factor loadings above .90. The items were then averaged to create a composite variable (mean 4.23, SD 1.79, alpha=.97).

#### Manipulation Checks

##### Perceptions of a Physician’s Technical Skills

To determine whether or not the manipulation of a physician’s technical skills was successful, 7 items were used to assess participants’ perceptions of this aspect (eg, *knowledgeable*, *competent*, and *skilled*). Participants were asked how well each of the 7 items described Dr Smith on a 7-point scale (1=very poorly, 7=very well). The items were averaged (mean 5.42, SD 1.34, alpha=.98). As predicted, participants assigned to conditions of high technical skills (mean 6.39, SD 0.75) perceived the physician to be more skilled technically compared with those assigned to conditions of moderate technical skills (mean 4.44, SD 1.06, *t*_483.47_=24.78 *, P*<.001).

##### Perceptions of a Physician’s Interpersonal Skills

Another set of 7 items was used to measure participants’ perceptions of Dr Smith’s interpersonal skills (eg, *Kind*, *Friendly*, and *Easy to talk to*) on a 7-point scale (1=very poorly, 7=very well; mean 5.26, SD 1.24, alpha=.96). As predicted, participants assigned to conditions of high interpersonal skills (mean 6.15, SD 0.79) perceived physicians to be more skilled interpersonally compared with those assigned to conditions of moderate interpersonal skills (mean 4.38, SD 0.95, *t*_520.51_=23.54, *P*<.001).

#### Control Variables

Current search for a physician. Participants were asked 2 questions to determine whether they were currently searching or recently intending to search for a primary care physician or back surgeon, dependent on the condition they were assigned to (eg, “How likely are you to try and find a new primary care physician or back surgeon in the next twelve months?”). They answered the questions on a 7-point scale (1=will definitely not; 7=will definitely; mean 2.88, SD 1.59).

##### Past Experience With a Physician

Participants were also asked 2 questions about their past experiences about looking for or having a primary care physician or back surgeon based on the physician type they were assigned to (eg, “Have you ever had a primary care physician or back surgeon?”, “Have you ever searched for a primary care physician or back surgeon?”). Participants answered either yes (1) or no (2) to both questions. For participants assigned to conditions involving a primary care physician, 76.6% (209/273) reported that they have had a primary care physician and 76.2% (208/273) reported that they have searched for a primary care physician. For participants assigned to conditions involving a back surgeon, only 5.2% (14/267) reported that they have had a back surgeon and 12.4% (33/267) reported that they have searched for a back surgeon.

##### Perceived Reliability of Ratings

Previous research has suggested that people may perceive the reliability of Web-based ratings differently [3,35], which, in turn, may affect their willingness to choose a physician. To control for the variation, participants were asked 1 question to assess the extent to which they consider the Web-based ratings reliable (ie, “To what extent do you consider the patient ratings are reliable measures of Dr Smith’s quality?”). The item was rated on a 7-point scale with the anchors 1=not reliable at all and 7=completely reliable (mean 5.14, SD 1.05).

### Data Analysis

We first examined whether data met the assumption on normality. For sample sizes greater than 300, an absolute skew value greater than 2 or an absolute kurtosis greater than 7 suggests data are non-normal [36]. The dependent variable of willingness to choose a physician has a relatively normal distribution, skewness=−.22, kurtosis=−1.08. Parametric tests were used to examine the research question and hypotheses.

We then conducted a 4-way (valence of technical skills × valence of interpersonal skills × physician type × orders of ratings) analysis of covariance (ANCOVA) on people’s willingness to choose a physician, controlling for current searching for a physician, past experience with a physician, and perceived reliability of Web-based ratings. As the order of ratings did not affect people’s willingness to choose a physician, *F*_1,521_=.017, *P*=.90, this factor was not examined further in subsequent analyses. After conducting the ANCOVA, a planned comparison *t* test was conducted to examine the research question on whether people place more weight on technical or interpersonal skills when selecting a physician. H1 and H2 were tested with tests of simple main effects. All analyses were run using SPSS Statistics version 25. The significance level to reject a null hypothesis was set to .05 for all analyses.

## Results

### Selection of a Physician in General

The research question concerns people’s willingness to choose a physician with higher ratings on one aspect than the other. The ANCOVA test suggests that the 2-way interaction between ratings of technical skills and ratings of interpersonal skills significantly affected people’s willingness to choose a physician, *F*_1,521_=30.42, *P*<.001, η_p_^2^=.06. A planned comparison *t* test was then conducted to further examine whether people are more willing to choose a physician with higher ratings on technical skills or interpersonal skills. The condition of high ratings on both skills was assigned a weight of 2; the condition of moderate ratings on both skills was assigned a weight of −2; the condition of high ratings on technical skills and moderate ratings on interpersonal skills was assigned a weight of 1; the condition of high ratings on interpersonal skills and moderate ratings on technical skills was assigned a weight of −1. The results suggested that people were significantly more likely to choose a physician with higher ratings on technical skills than on interpersonal skills (mean 4.79, SD 1.28) compared with a physician with higher ratings on interpersonal skills than on technical skills (mean 3.06, SD 1.55, *t*_369.96_=22.36, *P*<.001, Cohen *d*=1.22).

### Importance of Technical Versus Interpersonal skills in Selection of Different Types of Physicians

The first hypothesis predicted that people had higher intention to choose a specialist who has higher ratings on technical skills than on interpersonal skills, compared with a primary care physician with the same ratings. A 3-way interaction among ratings of technical skills, ratings of interpersonal skills, and physician type was not significant, *F*_1,521_=3.68, *P*=.06, η_p_^2^=.01. A posthoc analysis was then conducted to test the simple main effects of physician types within the interaction of technical and interpersonal skills. As predicted, participants were more willing to choose a specialist with higher ratings on technical skills than on interpersonal skills (mean 5.07, SD 1.38) compared with a primary care physician with the same ratings (mean 4.50, SD 1.36), *F*_1,521_=5.34, *P*=.02. Hence, H1 was supported. Table 2 presents means and SDs of the measured variable for all conditions.

**Table 2:** Means and SDs of willingness to choose a physician (N=540).

Variables	Primary care physician				Specialist			
	High ratings on technical skills		Moderate ratings on technical skills		High ratings on technical skills		Moderate ratings on technical skills	
	IS^a^, High	IS, Moderate	IS, High	IS, Moderate	IS, High	IS, Moderate	IS, High	IS, Moderate
Willingness to choose a physician, mean (SD)	6.07 (0.72)	4.50 (1.36)	3.09 (1.40)	3.26 (1.42)	5.91 (0.85)	5.07 (1.38)	3.06 (1.39)	2.94 (1.41)

^a^IS: interpersonal skills

The second hypothesis proposed that people were more willing to choose a primary care physician who has higher ratings on interpersonal skills than on technical skills, compared with a specialist with the same ratings. Contradictory to the prediction, the test of simple main effects suggested that people did not differ in their willingness to select a primary care physician (mean 3.09, SD 1.40) and a specialist (mean 3.06, SD 1.39) when the physician had higher ratings on interpersonal skills than on technical skills, *F*_1, 521_=0.013, *P*=.91. Therefore, H2 was not supported.

## Discussion

### Principal Findings

Patients are increasingly empowered in this rapidly changing health care landscape. With the access to physician reviews on the Web, patients are taking a more active role in their selection of physicians. Physicians and patients have different attitudes toward reviews provided on PRWs [6]. Physicians tend to question the accuracy of Web-based reviews and view them as a threat to their reputations [4], whereas patients generally have a favorable attitude and would consult these reviews in their choice of physicians [3]. It is thus imperative to understand how Web-based reviews affect patients’ perceptions and choice of physicians, which may help patients and health professionals have a better understanding of the role of PRWs in health consumerism.

Specifically, this study took the initiative to examine if Web-based physician ratings affect patients’ selection of primary care physicians and specialists differently. We investigated how Web-based reviews focusing on physicians’ technical and interpersonal skills affect people’s intention to select different types of physicians. The results showed that people were more willing to choose a physician with higher ratings on technical skills than on interpersonal skills compared with a physician with higher ratings on interpersonal skills than on technical skills. Furthermore, people perceived technical skills as more important and were more willing to compromise on interpersonal skills in their choice of a specialist compared with a primary care physician.

This study contributes to previous research on physician selection via PRWs by experimentally testing one’s preference for a physician who is high on technical skills versus interpersonal skills. Apart from previous research that relied on survey measures to assess patient’s preference for a physician’s skills, little work has experimentally tested the preference. By presenting patients with a mockup physician review site and a medical care vignette, we are able to aid the patients in imagining themselves in a medical situation and thus make their preferences more accessible. Before this study, it was unclear how specific factors such as rating categories or physician characteristics may affect people’s choice of physicians on PRWs [14]. With an experimental design, physician types and rating categories could be separately operationalized and directly compared to examine their role in people’s choice of physicians.

This study provides insight into understanding the impact of Web-based ratings on people’s physician selection. Beyond valence of Web-based reviews examined in previous research [10], this study investigated how ratings of different domains could affect people’s choice of physicians. The results suggested that patients tend to place more weight on technical skills than interpersonal skills when they choose physicians, regardless of physician types. Although previous research presented mixed findings on the relative importance of technical and interpersonal skills in people’s physician selection [12,15,16], this study found strong support for the greater importance of technical skills over interpersonal skills. It appears that sophisticated interpersonal skills cannot make up for the lack of medical competence. Therefore, having strong interpersonal skills, although still important, does not make a physician more competitive in the health market unless the physician is also technically competent. In fact, a post hoc analysis provided further evidence by showing that people did not differ in their willingness to choose a physician with high or moderate ratings on interpersonal skills, if the physician has mediocre ratings on technical skills.

Although technical skills, in general, are more valued than interpersonal skills in patients’ choice of physicians, the relative importance of these 2 skills may differ as a function of physician types [3]. It was unclear whether patients place different weightage on technical and interpersonal skills when choosing different types of physicians. To fill the gap, this study employed a controlled experiment to investigate this matter in the context of PRWs. As a primary care physician usually serves as the first check-up point before patients’ visit to a specialist who diagnoses and treats more complex problems, patients tend to expect more technical skills from a specialist compared with a primary care physician. Consistent with this prediction, when people were asked to make tradeoffs between a physician’s technical and interpersonal skills, they were more willing to compromise on interpersonal skills in their selection of a specialist compared with a primary care physician. Contradictory to our prediction, patients did not seem to value interpersonal skills more in their selection of primary care physicians versus specialists. Although many patients want to establish long-term relationships with their primary care physicians and value interpersonal rapport, they may set up high standards for primary care physicians’ technical skills as well. Interpersonal skills, to a certain extent, might be secondary to technical skills when people choose primary care physicians. After all, the cost of misdiagnosis or mistreatment is tremendous and may lead to irreversible consequences on patients’ health. The impact of ineffective interpersonal skills on a patient’s health seems to be less severe. Therefore, patients may take into account a primary care physician’s interpersonal competence only if this physician meets the high standards for technical skills.

### Practical Implications

These results have practical implications for physicians who have profiles on PRWs. Given that patients value technical skills over interpersonal skills, physicians who are confident with their technical skills should try to highlight this aspect in their Web presence. For instance, quite a few medical sites allow physicians to include self-descriptions or video biographies, which can serve as important venues to promote physicians’ technical skills [37]. Physicians should take advantage of these channels to advocate their technical skills. Moreover, research has shown that patients and physicians tend to have different attitudes toward PRWs. Patients are generally in favor of using this service, whereas physicians have some legitimate concerns over these sites [6]. If some patients present biased opinions about a physician’s technical skills, this may mislead other patients and harm the physician’s reputation. To mitigate the influence of biased reviews, PRWs may consider providing both parties (ie, patients and physicians) equal opportunities to present their opinions. For example, PRWs could expand physicians’ profile sections by allowing them to post multimedia contents, such as photos and videos of their work. In addition, physicians should be offered the option to respond to patient ratings and reviews on PRWs.

PRWs provide patients aggregated ratings on physicians’ technical and interpersonal skills, which could be indicative of physicians’ qualities and thus affect the patient choice of physicians. Although people consider ratings on both skills, ratings on technical competence, such as diagnosis and treatment, are given more weightage when choosing physicians, regardless of physician types. Therefore, PRWs could prioritize this skill set by providing more nuanced rating categories on technical skills.

### Limitations

This study has several limitations that point to directions for future research. First, because previous research suggests that only a small proportion of reviews on PRWs are negative [7,38], this study did not include negative ratings. Although we deliberately excluded negative ratings to represent the reality of PRWs, it would still be worthwhile to learn how negative ratings may affect people’s choice of physicians. In particular, negativity effects may take place such that patients are more impacted by negative ratings than positive ones on their selection of physicians. Under such circumstances, people may not be willing to choose a technically skilled physician who receives negative feedback on interpersonal skills.

Second, this study examined the impact of numerical ratings, but not narrative comments, on patients’ willingness to choose physicians. Although patients’ evaluations are primarily displayed in the format of aggregated numerical ratings on PRWs, many portals also allow patients to leave narrative comments to detail their satisfaction and dissatisfaction. Aggregated numerical ratings tend to provide patients a holistic view of physicians and the services they provide. Narrative comments, on the contrary, can capture more detailed and nuanced feedback that is not reflected in structured rating systems. A direction for future research is to investigate how numerical ratings and narrative comments work together to affect people’s willingness to choose a physician, especially if 2 sources present contradictory information.

Third, this study focused on rating categories pertaining to a physician’s technical and interpersonal skills. In selection of a physician, patients take into account many considerations beyond a physician’s qualifications. For example, previous research has found that management practices such as punctuality and staff quality are also considered in patients’ choice of physicians [31,39]. Besides reviewing a physician’s qualifications, many PRWs also include rating categories on management practices. Future research should look into these aspects in addition to a physician’s skills.

Fourth, despite a wide range of specialties, this study operationalized a specialist to be a back surgeon. However, it is likely that patients use different selection criteria for specialists of different types. Under certain circumstances, patients may place more weightage on a specialist’s interpersonal skills than technical skills (eg, visiting a gynecologist for a check-up). Future research thus needs to examine if the influence of Web-based ratings on physician choice differs as a function of physician specialties and medical conditions.

Finally, a patient’s willingness to choose a physician is influenced by a variety of factors beyond numerical ratings displayed on PRWs. For example, demographic information of a physician (eg, sex and age) and environmental factors (eg, office location) should be taken into account when examining patients’ choice of physicians. Another direction for future research is to explore underlying mechanisms, especially perceptual processes, through which physician types and patient reviews affect people’s choice of physicians.

### Conclusions

Patients increasingly seek information on the Web when looking for health care providers. The recent growth of PRWs has resulted in efforts to investigate how these platforms affect patients’ health decision making. This study sheds light on this matter by examining how Web-based ratings on a physician’s technical and interpersonal skills may affect people’s willingness to choose a primary care physician versus a specialist. The results suggest that patients value physicians’ technical skills more than their interpersonal skills when they select physicians. Patients are more willing to make a compromise on a physician’s interpersonal skills than technical skills in their choice of specialists compared with primary care physicians. Given the importance of technical skills in people’s choice of physicians, physicians who are confident in their technical skills should make efforts to promote such skills in their Web-based profiles. Carriers of PRWs should enhance the functionality of these platforms by allowing the upload of multimedia contents that physicians could use to deliver a strong Web presence. In addition, PRWs should enable physicians to respond to patients’ reviews if this function is not made available on platforms. Patients, as the primary users of PRWs, need to be aware of their impact on other users and be more responsible when leaving ratings about their physicians on the Web.

## Abbreviations

ANCOVA: analysis of covariance
PRW: physician review website

## Multimedia Appendix 1

CONSORT‐EHEALTH checklist (V 1.6.1).
