# Supplementary material for: The Impact of Web-Based Ratings on Patient Choice of a Primary Care Physician Versus a Specialist: Randomized Controlled Experiment
Source: J Med Internet Res. 2019 Jun 28;21(6):e11188. doi: 10.2196/11188 (PMC6625218; doi:10.2196/11188)
Supplement: Supplementary file 1 [file jmir_v21i6e11188_app1.pdf]

CONSORT-EHEALTH (V 1.6.1) - Submission/Publication Form

The CONSORT-EHEALTH checklist is intended for authors of randomized trials evaluating web-based and Internet-based applications/interventions, including mobile interventions, electronic games (incl multiplayer games), social media, certain telehealth applications, and other interactive and/or networked electronic applications. Some of the items (e.g. all subitems under item 5 - description of the intervention) may also be applicable for other study designs.

The goal of the CONSORT EHEALTH checklist and guideline is to be
a) a guide for reporting for authors of RCTs,
b) to form a basis for appraisal of an ehealth trial (in terms of validity)

CONSORT-EHEALTH items/subitems are MANDATORY reporting items for studies published in the Journal of Medical Internet Research and other journals / scientific societies endorsing the checklist.

Items numbered 1., 2., 3., 4a., 4b etc are original CONSORT or CONSORT-NPT (non-pharmacologic treatment) items.
Items with Roman numerals (i., ii, iii, iv etc.) are CONSORT-EHEALTH extensions/clarifications.

As the CONSORT-EHEALTH checklist is still considered in a formative stage, we would ask that you also RATE ON A SCALE OF 1-5 how important/useful you feel each item is FOR THE PURPOSE OF THE CHECKLIST and reporting guideline (optional).

Mandatory reporting items are marked with a red *.
In the textboxes, either copy & paste the relevant sections from your manuscript into this form - please include any quotes from your manuscript in QUOTATION MARKS,
or answer directly by providing additional information not in the manuscript, or elaborating on why the item was not relevant for this study.

YOUR ANSWERS WILL BE PUBLISHED AS A SUPPLEMENTARY FILE TO YOUR PUBLICATION IN JMIR AND ARE CONSIDERED PART OF YOUR PUBLICATION (IF ACCEPTED).
Please fill in these questions diligently. Information will not be copyedited, so please use proper spelling and grammar, use correct capitalization, and avoid abbreviations.

DO NOT FORGET TO SAVE AS PDF _AND_ CLICK THE SUBMIT BUTTON SO YOUR ANSWERS ARE IN OUR DATABASE !!!

Citation Suggestion (if you append the pdf as Appendix we suggest to cite this paper in the caption):
Eysenbach G, CONSORT-EHEALTH Group
CONSORT-EHEALTH: Improving and Standardizing Evaluation Reports of Web-based and Mobile Health Interventions
J Med Internet Res 2011;13(4):e126
URL: http://www.jmir.org/2011/4/e126/
doi: 10.2196/jmir.1923
PMID: 22209829

* Required

Your name *

First Last

Siyue Li

Primary Affiliation (short), City, Country *

University of Toronto, Toronto, Canada

Zhejiang University, Hangzhou, China

Your e-mail address *

abc@gmail.com

siyueaprilli@gmail.com

Title of your manuscript *

Provide the (draft) title of your manuscript.

The Impact of Web-Based Ratings on Patient Choice of a Primary Care Physician Versus a Specialist: Randomized Controlled Experiment

Name of your App/Software/Intervention *

If there is a short and a long/alternate name, write the short name first and add the long name in brackets.

Physician rating websites

Evaluated Version (if any)

e.g. "V1", "Release 2017-03-01", "Version 2.0.27913"

Your answer

Language(s) *

What language is the intervention/app in? If multiple languages are available, separate by comma (e.g. "English, French")

English

URL of your Intervention Website or App

e.g. a direct link to the mobile app on app in appstore (itunes, Google Play), or URL of the website. If the intervention is a DVD or hardware, you can also link to an Amazon page.

Your answer

URL of an image/screenshot (optional)

Your answer

Accessibility *

Can an enduser access the intervention presently?

- ○ access is free and open
- ○ access only for special usergroups, not open
- ○ access is open to everyone, but requires payment/subscription/in-app purchases
- ◉ app/intervention no longer accessible
- ○ Other:

Primary Medical Indication/Disease/Condition *

e.g. "Stress", "Diabetes", or define the target group in brackets after the condition, e.g. "Autism (Parents of children with)", "Alzheimers (Informal Caregivers of)"

Primary care physician, specialist

Primary Outcomes measured in trial *

comma-separated list of primary outcomes reported in the trial

Willingness to choose a physician's servic

Secondary/other outcomes

Are there any other outcomes the intervention is expected to affect?

Your answer

Recommended "Dose" *

What do the instructions for users say on how often the app should be used?

- ○ Approximately Daily
- ○ Approximately Weekly
- ○ Approximately Monthly
- ○ Approximately Yearly
- ○ "as needed"
- ◉ Other: not applicable

Approx. Percentage of Users (starters) still using the app as recommended after 3 months *

- (•) unknown / not evaluated
- ( ) 0-10%
- ( ) 11-20%
- ( ) 21-30%
- ( ) 31-40%
- ( ) 41-50%
- ( ) 51-60%
- ( ) 61-70%
- ( ) 71%-80%
- ( ) 81-90%
- ( ) 91-100%
- ( ) Other:

Overall, was the app/intervention effective? *

- ( ) yes: all primary outcomes were significantly better in intervention group vs control
- (•) partly: SOME primary outcomes were significantly better in intervention group vs control
- ( ) no statistically significant difference between control and intervention
- ( ) potentially harmful: control was significantly better than intervention in one or more outcomes
- ( ) inconclusive: more research is needed
- ( ) Other:

Article Preparation Status/Stage *

At which stage in your article preparation are you currently (at the time you fill in this form)

- ( ) not submitted yet - in early draft status
- ( ) not submitted yet - in late draft status, just before submission
- ( ) submitted to a journal but not reviewed yet
- ( ) submitted to a journal and after receiving initial reviewer comments
- (•) submitted to a journal and accepted, but not published yet
- ( ) published
- ( ) Other:

Journal *

If you already know where you will submit this paper (or if it is already submitted), please provide the journal name (if it is not JMIR, provide the journal name under "other")

- ○ not submitted yet / unclear where I will submit this
- ◉ Journal of Medical Internet Research (JMIR)
- ○ JMIR mHealth and UHealth
- ○ JMIR Serious Games
- ○ JMIR Mental Health
- ○ JMIR Public Health
- ○ JMIR Formative Research
- ○ Other JMIR sister journal
- ○ Other:

Is this a full powered effectiveness trial or a pilot/feasibility trial? *

- ○ Pilot/feasibility
- ◉ Fully powered

Manuscript tracking number *

If this is a JMIR submission, please provide the manuscript tracking number under "other" (The ms tracking number can be found in the submission acknowledgement email, or when you login as author in JMIR. If the paper is already published in JMIR, then the ms tracking number is the four-digit number at the end of the DOI, to be found at the bottom of each published article in JMIR)

- ○ no ms number (yet) / not (yet) submitted to / published in JMIR
- ◉ Other: 11188

TITLE AND ABSTRACT

1a) TITLE: Identification as a randomized trial in the title

1a) Does your paper address CONSORT item 1a? *

I.e does the title contain the phrase "Randomized Controlled Trial"? (if not, explain the reason under "other")

- (•) yes
- ( ) Other:

1a-i) Identify the mode of delivery in the title

Identify the mode of delivery. Preferably use “web-based” and/or “mobile” and/or “electronic game” in the title. Avoid ambiguous terms like “online”, “virtual”, “interactive”. Use “Internet-based” only if Intervention includes non-web-based Internet components (e.g. email), use “computer-based” or “electronic” only if offline products are used. Use “virtual” only in the context of “virtual reality” (3-D worlds). Use “online” only in the context of “online support groups”. Complement or substitute product names with broader terms for the class of products (such as “mobile” or “smart phone” instead of “iphone”), especially if the application runs on different platforms.

	1	2	3	4	5	
subitem not at all important	( )	( )	( )	( )	( )	essential

Does your paper address subitem 1a-i? *

Copy and paste relevant sections from manuscript title (include quotes in quotation marks "like this" to indicate direct quotes from your manuscript), or elaborate on this item by providing additional information not in the ms, or briefly explain why the item is not applicable/relevant for your study

"Web-Based Ratings"

1a-ii) Non-web-based components or important co-interventions in title

Mention non-web-based components or important co-interventions in title, if any (e.g., “with telephone support”).

	1	2	3	4	5	
subitem not at all important	○	○	○	○	○	essential

Does your paper address subitem 1a-ii?

Copy and paste relevant sections from manuscript title (include quotes in quotation marks "like this" to indicate direct quotes from your manuscript), or elaborate on this item by providing additional information not in the ms, or briefly explain why the item is not applicable/relevant for your study

"Patient Choice of a Primary Care Physician Versus a Specialist"

1a-iii) Primary condition or target group in the title

Mention primary condition or target group in the title, if any (e.g., “for children with Type I Diabetes”) Example: A Web-based and Mobile Intervention with Telephone Support for Children with Type I Diabetes: Randomized Controlled Trial

	1	2	3	4	5	
subitem not at all important	○	○	○	○	○	essential

Does your paper address subitem 1a-iii? *

Copy and paste relevant sections from manuscript title (include quotes in quotation marks "like this" to indicate direct quotes from your manuscript), or elaborate on this item by providing additional information not in the ms, or briefly explain why the item is not applicable/relevant for your study

"Patient choice"

1b) ABSTRACT: Structured summary of trial design, methods, results, and conclusions

NPT extension: Description of experimental treatment, comparator, care providers, centers, and blinding status.

1b-i) Key features/functionalities/components of the intervention and comparator in the METHODS section of the ABSTRACT

Mention key features/functionalities/components of the intervention and comparator in the abstract. If possible, also mention theories and principles used for designing the site. Keep in mind the needs of systematic reviewers and indexers by including important synonyms. (Note: Only report in the abstract what the main paper is reporting. If this information is missing from the main body of text, consider adding it)

	1	2	3	4	5	
subitem not at all important	○	○	○	○	○	essential

Does your paper address subitem 1b-i? *

Copy and paste relevant sections from the manuscript abstract (include quotes in quotation marks "like this" to indicate direct quotes from your manuscript), or elaborate on this item by providing additional information not in the ms, or briefly explain why the item is not applicable/relevant for your study

"A 2x2x2x2 between-subjects experiment was conducted. Over 600 participants were recruited through a crowdsourcing website and randomly assigned to view a mockup physician review Web page that contained information on a physician's basic information and patients' ratings. After reviewing the Web page, participants were asked to complete a survey on their perceptions of the physician and willingness to seek health care from the physician. "

1b-ii) Level of human involvement in the METHODS section of the ABSTRACT

Clarify the level of human involvement in the abstract, e.g., use phrases like "fully automated" vs. "therapist/nurse/care provider/physician-assisted" (mention number and expertise of providers involved, if any). (Note: Only report in the abstract what the main paper is reporting. If this information is missing from the main body of text, consider adding it)

	1	2	3	4	5	
subitem not at all important	○	○	○	○	○	essential

Does your paper address subitem 1b-ii?

Copy and paste relevant sections from the manuscript abstract (include quotes in quotation marks "like this" to indicate direct quotes from your manuscript), or elaborate on this item by providing additional information not in the ms, or briefly explain why the item is not applicable/relevant for your study

fully automated

1b-iii) Open vs. closed, web-based (self-assessment) vs. face-to-face assessments in the METHODS section of the ABSTRACT

Mention how participants were recruited (online vs. offline), e.g., from an open access website or from a clinic or a closed online user group (closed usergroup trial), and clarify if this was a purely web-based trial, or there were face-to-face components (as part of the intervention or for assessment). Clearly say if outcomes were self-assessed through questionnaires (as common in web-based trials). Note: In traditional offline trials, an open trial (open-label trial) is a type of clinical trial in which both the researchers and participants know which treatment is being administered. To avoid confusion, use “blinded” or “unblinded” to indicated the level of blinding instead of “open”, as “open” in web-based trials usually refers to “open access” (i.e. participants can self-enrol). (Note: Only report in the abstract what the main paper is reporting. If this information is missing from the main body of text, consider adding it)

	1	2	3	4	5	
subitem not at all important	○	○	○	○	○	essential

Does your paper address subitem 1b-iii?

Copy and paste relevant sections from the manuscript abstract (include quotes in quotation marks "like this" to indicate direct quotes from your manuscript), or elaborate on this item by providing additional information not in the ms, or briefly explain why the item is not applicable/relevant for your study

"Over 600 participants were recruited through a crowdsourcing website and randomly assigned to view a mockup physician review Web page that contained information on a physician’s basic information and patients’ ratings. After reviewing the Web page, participants were asked to complete a survey on their perceptions of the physician and willingness to seek health care from the physician. "

1b-iv) RESULTS section in abstract must contain use data

Report number of participants enrolled/assessed in each group, the use/uptake of the intervention (e.g., attrition/adherence metrics, use over time, number of logins etc.), in addition to primary/secondary outcomes. (Note: Only report in the abstract what the main paper is reporting. If this information is missing from the main body of text, consider adding it)

	1	2	3	4	5	
subitem not at all important	○	○	○	○	○	essential

Does your paper address subitem 1b-iv?

Copy and paste relevant sections from the manuscript abstract (include quotes in quotation marks "like this" to indicate direct quotes from your manuscript), or elaborate on this item by providing additional information not in the ms, or briefly explain why the item is not applicable/relevant for your study

"The results showed that participants were more willing to choose a physician with higher ratings on technical skills than on interpersonal skills compared with a physician with higher ratings on interpersonal skills than on technical skills, $t_{369.96}=22.36$, $P<.001$, Cohen d=1.22. In the selection of different types of physicians, patients were more likely to choose a specialist with higher ratings on technical skills than on interpersonal skills, compared with a primary care physician with the same ratings, $F_{1,521}=5.34$, $P=.021$. "

1b-v) CONCLUSIONS/DISCUSSION in abstract for negative trials

Conclusions/Discussions in abstract for negative trials: Discuss the primary outcome - if the trial is negative (primary outcome not changed), and the intervention was not used, discuss whether negative results are attributable to lack of uptake and discuss reasons. (Note: Only report in the abstract what the main paper is reporting. If this information is missing from the main body of text, consider adding it)

	1	2	3	4	5	
subitem not at all important	○	○	○	○	○	essential

Does your paper address subitem 1b-v?

Copy and paste relevant sections from the manuscript abstract (include quotes in quotation marks "like this" to indicate direct quotes from your manuscript), or elaborate on this item by providing additional information not in the ms, or briefly explain why the item is not applicable/relevant for your study

"The findings suggest that people place more weight on technical skills than interpersonal skills in their selection of a physician based on their ratings on the Web. Specifically, people are more likely to make a compromise on interpersonal skills in their choice of a specialist compared with a primary care physician. This study emphasizes the importance of examining Web-based physician ratings in a more nuanced way in relation to the selection of different types of physicians."

INTRODUCTION

2a) In INTRODUCTION: Scientific background and explanation of rationale

2a-i) Problem and the type of system/solution

Describe the problem and the type of system/solution that is object of the study: intended as stand-alone intervention vs. incorporated in broader health care program? Intended for a particular patient population? Goals of the intervention, e.g., being more cost-effective to other interventions, replace or complement other solutions? (Note: Details about the intervention are provided in “Methods” under 5)

	1	2	3	4	5	
subitem not at all important	○	○	○	○	○	essential

Does your paper address subitem 2a-i? *

Copy and paste relevant sections from the manuscript (include quotes in quotation marks "like this" to indicate direct quotes from your manuscript), or elaborate on this item by providing additional information not in the ms, or briefly explain why the item is not applicable/relevant for your study

The objectives of this study were to examine (1) how patients weigh ratings on a physician’s technical skills and interpersonal skills in their selection of physicians and (2) whether and how people’s choice of a primary care physician versus a specialist is affected differently by Web-based ratings.

2a-ii) Scientific background, rationale: What is known about the (type of) system

Scientific background, rationale: What is known about the (type of) system that is the object of the study (be sure to discuss the use of similar systems for other conditions/diagnoses, if appropiate), motivation for the study, i.e. what are the reasons for and what is the context for this specific study, from which stakeholder viewpoint is the study performed, potential impact of findings [2]. Briefly justify the choice of the comparator.

	1	2	3	4	5	
subitem not at all important	○	○	○	○	○	essential

Does your paper address subitem 2a-ii? *

Copy and paste relevant sections from the manuscript (include quotes in quotation marks "like this" to indicate direct quotes from your manuscript), or elaborate on this item by providing additional information not in the ms, or briefly explain why the item is not applicable/relevant for your study

"With the growing popularity of PRWs, researchers have recently begun to examine the role of these websites in people’s health decision making [5-8]. The scholarship on PRWs covers a wide range of topics, including but not limited to demographics of website users, structures of the portals, patterns of website usage, and content of reviews [9-11]. Technical skills and interpersonal skills reside at the core of a physician’s qualifications and are commonly rated on PRWs [12,13]. However, little is known about how ratings on these different aspects of a physician may affect patients’ choice differently [14]. Previous research presents mixed results on how people set the priority of technical and interpersonal skills in physician selection [15,16]. Therefore, the first goal of this study was to examine how patients prioritize technical and interpersonal skills in their physician selection based on ratings on PRWs.

The second objective of this study was to examine whether and how people’s choice of a primary care physician versus a specialist is affected differently by Web-based ratings. In the United States, patients are allowed greater autonomy to choose their primary care physicians compared with specialists. As a result, significantly more research focuses on people’s selection of primary care physicians than physicians of other types [16-18]. Nowadays, however, patients are more involved in the choice of specialists in part because of the easy access of health information on the Web. Given that primary care physicians and specialists take on different roles in health care, patients may apply different criteria to select different types of physicians [3]. In this study, we specifically examine how ratings on a physician’s technical and interpersonal skills may affect patients’ choice of primary care physicians and specialists differently. "

2b) In INTRODUCTION: Specific objectives or hypotheses

Does your paper address CONSORT subitem 2b? *

Copy and paste relevant sections from the manuscript (include quotes in quotation marks "like this" to indicate direct quotes from your manuscript), or elaborate on this item by providing additional information not in the ms, or briefly explain why the item is not applicable/relevant for your study

"RQ1: Are people more willing to choose a physician with higher ratings on technical skills than on interpersonal skills, or a physician with higher ratings on interpersonal skills than on technical skills?

H1: People are more willing to choose a specialist who has higher ratings on technical skills than on interpersonal skills, compared with a primary care physician who has the same ratings.

H2: People are more willing to choose a primary care physician who has higher ratings on interpersonal skills than on technical skills, compared with a specialist who has the same ratings."

METHODS

3a) Description of trial design (such as parallel, factorial) including allocation ratio

Does your paper address CONSORT subitem 3a? *

Copy and paste relevant sections from the manuscript (include quotes in quotation marks "like this" to indicate direct quotes from your manuscript), or elaborate on this item by providing additional information not in the ms, or briefly explain why the item is not applicable/relevant for your study

"To investigate the proposed research question and hypotheses, a 2 (ratings on communication skills: high versus moderate) x 2 (ratings on technical skills: high versus moderate) x 2 (physician specialty: primary care physician versus specialist) x 2 (order of ratings: interpersonal skill first versus technical skills first) between-subjects factorial design was employed. "

3b) Important changes to methods after trial commencement (such as eligibility criteria), with reasons

Does your paper address CONSORT subitem 3b? *

Copy and paste relevant sections from the manuscript (include quotes in quotation marks "like this" to indicate direct quotes from your manuscript), or elaborate on this item by providing additional information not in the ms, or briefly explain why the item is not applicable/relevant for your study

No changes to methods were made after trail commencement.

3b-i) Bug fixes, Downtimes, Content Changes

Bug fixes, Downtimes, Content Changes: ehealth systems are often dynamic systems. A description of changes to methods therefore also includes important changes made on the intervention or comparator during the trial (e.g., major bug fixes or changes in the functionality or content) (5-iii) and other “unexpected events” that may have influenced study design such as staff changes, system failures/downtimes, etc. [2].

	1	2	3	4	5	
subitem not at all important	○	○	○	○	○	essential

Does your paper address subitem 3b-i?

Copy and paste relevant sections from the manuscript (include quotes in quotation marks "like this" to indicate direct quotes from your manuscript), or elaborate on this item by providing additional information not in the ms, or briefly explain why the item is not applicable/relevant for your study

No changes were made afterwards.

4a) Eligibility criteria for participants

Does your paper address CONSORT subitem 4a? *

Copy and paste relevant sections from the manuscript (include quotes in quotation marks "like this" to indicate direct quotes from your manuscript), or elaborate on this item by providing additional information not in the ms, or briefly explain why the item is not applicable/relevant for your study

"A total of 608 participants completed the Web-based experiment. Participants were recruited via the crowdsourcing website, Amazon’s Mechanical Turk (mTurk), and compensated for their time. We excluded people who failed the attention checks (n=26) and those who spent no time (n=1) or less than 5 seconds on the Web page (n=41). "

4a-i) Computer / Internet literacy

Computer / Internet literacy is often an implicit “de facto” eligibility criterion - this should be explicitly clarified.

	1	2	3	4	5	
subitem not at all important	○	○	○	○	○	essential

Does your paper address subitem 4a-i?

Copy and paste relevant sections from the manuscript (include quotes in quotation marks "like this" to indicate direct quotes from your manuscript), or elaborate on this item by providing additional information not in the ms, or briefly explain why the item is not applicable/relevant for your study

Participants' use of physician review websites was measured and entered as a control variable.

4a-ii) Open vs. closed, web-based vs. face-to-face assessments:

Open vs. closed, web-based vs. face-to-face assessments: Mention how participants were recruited (online vs. offline), e.g., from an open access website or from a clinic, and clarify if this was a purely web-based trial, or there were face-to-face components (as part of the intervention or for assessment), i.e., to what degree got the study team to know the participant. In online-only trials, clarify if participants were quasi-anonymous and whether having multiple identities was possible or whether technical or logistical measures (e.g., cookies, email confirmation, phone calls) were used to detect/prevent these.

	1	2	3	4	5	
subitem not at all important	○	○	○	○	○	essential

Does your paper address subitem 4a-ii? *

Copy and paste relevant sections from the manuscript (include quotes in quotation marks "like this" to indicate direct quotes from your manuscript), or elaborate on this item by providing additional information not in the ms, or briefly explain why the item is not applicable/relevant for your study

Participants were anonymous in this online-only trial.

4a-iii) Information giving during recruitment

Information given during recruitment. Specify how participants were briefed for recruitment and in the informed consent procedures (e.g., publish the informed consent documentation as appendix, see also item X26), as this information may have an effect on user self-selection, user expectation and may also bias results.

	1	2	3	4	5	
subitem not at all important	○	○	○	○	○	essential

Does your paper address subitem 4a-iii?

Copy and paste relevant sections from the manuscript (include quotes in quotation marks "like this" to indicate direct quotes from your manuscript), or elaborate on this item by providing additional information not in the ms, or briefly explain why the item is not applicable/relevant for your study

Each participant read and sign the consent form online before conducting the study.

4b) Settings and locations where the data were collected

Does your paper address CONSORT subitem 4b? *

Copy and paste relevant sections from the manuscript (include quotes in quotation marks "like this" to indicate direct quotes from your manuscript), or elaborate on this item by providing additional information not in the ms, or briefly explain why the item is not applicable/relevant for your study

Data were collected online. Participants completed the study using a digital device/at a location of their choice.

4b-i) Report if outcomes were (self-)assessed through online questionnaires

Clearly report if outcomes were (self-)assessed through online questionnaires (as common in web-based trials) or otherwise.

	1	2	3	4	5	
subitem not at all important	○	○	○	○	○	essential

Does your paper address subitem 4b-i? *

Copy and paste relevant sections from the manuscript (include quotes in quotation marks "like this" to indicate direct quotes from your manuscript), or elaborate on this item by providing additional information not in the ms, or briefly explain why the item is not applicable/relevant for your study

Data were self-reports through online questionnaires.

4b-ii) Report how institutional affiliations are displayed

Report how institutional affiliations are displayed to potential participants [on ehealth media], as affiliations with prestigious hospitals or universities may affect volunteer rates, use, and reactions with regards to an intervention.(Not a required item – describe only if this may bias results)

	1	2	3	4	5	
subitem not at all important	○	○	○	○	○	essential

Does your paper address subitem 4b-ii?

Copy and paste relevant sections from the manuscript (include quotes in quotation marks "like this" to indicate direct quotes from your manuscript), or elaborate on this item by providing additional information not in the ms, or briefly explain why the item is not applicable/relevant for your study

Institutional affiliations were not displayed in the stimuli or survey.

5) The interventions for each group with sufficient details to allow replication, including how and when they were actually administered

5-i) Mention names, credential, affiliations of the developers, sponsors, and owners

Mention names, credential, affiliations of the developers, sponsors, and owners [6] (if authors/evaluators are owners or developer of the software, this needs to be declared in a “Conflict of interest” section or mentioned elsewhere in the manuscript).

	1	2	3	4	5	
subitem not at all important	○	○	○	○	○	essential

Does your paper address subitem 5-i?

Copy and paste relevant sections from the manuscript (include quotes in quotation marks "like this" to indicate direct quotes from your manuscript), or elaborate on this item by providing additional information not in the ms, or briefly explain why the item is not applicable/relevant for your study

"Following consent, participants were presented a cover story to read. On the basis of the type of physician that they were assigned to, participants were asked to imagine themselves in a situation looking for either a primary care physician or a surgeon. The vignette about a primary care physician depicted a situation that the participant recently moved to a new city and was in need of a new primary care physician. Owing to a lack of input from family members and friends, they decided to search for primary care physicians on PRWs. The vignette about a surgeon described a situation in which the participant had lasting back pains. The primary care physician suspected that the patient may need spinal surgery and provided a list of surgeons to choose from. The participant decided to search for the recommended surgeons on PRWs. After reading through the scenario and imaging themselves in the described situation, each participant was directed to a physician review page to learn about the physician.

A total of 16 physician review pages were developed for this study (see Figure 1). The top part of each page listed basic information about a physician, including the physician’s name (Dr J Smith), the specialty (family medicine or surgeon), and information on new patient acceptance (accepting new patients). To manipulate the type of a physician, half of the Web pages listed the physician’s specialty as family medicine and the other half described the physician as a surgeon.

Each page contained 4 aggregated rating categories about Dr Smith, including 2 items on technical skills (“My doctor accurately diagnosed my problem” and “My doctor effectively treated my problem”) and 2 on interpersonal skills (“My doctor was caring” and “My doctor spent enough time with me”). Past research has suggested that a physician’s skills on diagnosis and treatment are among the most important considerations when selecting a physician [32]. In addition, a physician’s personal manner as well as time spent with a patient are critical to a patient’s satisfaction on the physician’s interpersonal skills [32,33]. These 4 categories frequently appear on PRWs [34] and thus are adopted in this study. To manipulate the valence of physician ratings, these rating categories were assigned different star ratings. Each rating category was presented in the form of aggregated ratings. In the conditions where a physician received high ratings on technical skills, the 2 items pertaining to technical skills were given 5/5-star ratings. In the conditions of moderate ratings on technical skills, the same items were assigned 3/5-star ratings. We chose to examine moderate ratings instead of low ratings in this study because

research suggests that low ratings are relatively uncommon on PRWs . The valence of a physician's interpersonal skills was manipulated in the same way. Furthermore, the rating categories were presented to participants in counterbalanced order to control for the impact of rating order effects. In half of the experimental conditions, the 2 rating categories on technical skills were displayed before the 2 categories on interpersonal skills. In the other half, ratings on technical skills were presented beneath the ratings on interpersonal skills. "

5-ii) Describe the history/development process

Describe the history/development process of the application and previous formative evaluations (e.g., focus groups, usability testing), as these will have an impact on adoption/use rates and help with interpreting results.

	1	2	3	4	5	
subitem not at all important	○	○	○	○	○	essential

Does your paper address subitem 5-ii?

Copy and paste relevant sections from the manuscript (include quotes in quotation marks "like this" to indicate direct quotes from your manuscript), or elaborate on this item by providing additional information not in the ms, or briefly explain why the item is not applicable/relevant for your study

We reviewed physician review websites before generating the stimuli.

5-iii) Revisions and updating

Revisions and updating. Clearly mention the date and/or version number of the application/intervention (and comparator, if applicable) evaluated, or describe whether the intervention underwent major changes during the evaluation process, or whether the development and/or content was "frozen" during the trial. Describe dynamic components such as news feeds or changing content which may have an impact on the replicability of the intervention (for unexpected events see item 3b).

	1	2	3	4	5	
subitem not at all important	○	○	○	○	○	essential

Does your paper address subitem 5-iii?

Copy and paste relevant sections from the manuscript (include quotes in quotation marks "like this" to indicate direct quotes from your manuscript), or elaborate on this item by providing additional information not in the ms, or briefly explain why the item is not applicable/relevant for your study

Not applicable. We examined physician rating websites in general, not a specific one.

5-iv) Quality assurance methods

Provide information on quality assurance methods to ensure accuracy and quality of information provided [1], if applicable.

	1	2	3	4	5	
subitem not at all important	○	○	○	○	○	essential

Does your paper address subitem 5-iv?

Copy and paste relevant sections from the manuscript (include quotes in quotation marks "like this" to indicate direct quotes from your manuscript), or elaborate on this item by providing additional information not in the ms, or briefly explain why the item is not applicable/relevant for your study

We used attention checks and manipulation checks. All measures were reliable.

5-v) Ensure replicability by publishing the source code, and/or providing screenshots/screen-capture video, and/or providing flowcharts of the algorithms used

Ensure replicability by publishing the source code, and/or providing screenshots/screen-capture video, and/or providing flowcharts of the algorithms used. Replicability (i.e., other researchers should in principle be able to replicate the study) is a hallmark of scientific reporting.

	1	2	3	4	5	
subitem not at all important	○	○	○	○	○	essential

Does your paper address subitem 5-v?

Copy and paste relevant sections from the manuscript (include quotes in quotation marks "like this" to indicate direct quotes from your manuscript), or elaborate on this item by providing additional information not in the ms, or briefly explain why the item is not applicable/relevant for your study

Screenshots of the stimuli were provided in the manuscript.

5-vi) Digital preservation

Digital preservation: Provide the URL of the application, but as the intervention is likely to change or disappear over the course of the years; also make sure the intervention is archived (Internet Archive, webcitation.org, and/or publishing the source code or screenshots/videos alongside the article). As pages behind login screens cannot be archived, consider creating demo pages which are accessible without login.

	1	2	3	4	5	
subitem not at all important	○	○	○	○	○	essential

Does your paper address subitem 5-vi?

Copy and paste relevant sections from the manuscript (include quotes in quotation marks "like this" to indicate direct quotes from your manuscript), or elaborate on this item by providing additional information not in the ms, or briefly explain why the item is not applicable/relevant for your study

Screenshots were provided.

5-vii) Access

Access: Describe how participants accessed the application, in what setting/context, if they had to pay (or were paid) or not, whether they had to be a member of specific group. If known, describe how participants obtained “access to the platform and Internet” [1]. To ensure access for editors/reviewers/readers, consider to provide a “backdoor” login account or demo mode for reviewers/readers to explore the application (also important for archiving purposes, see vi).

	1	2	3	4	5	
subitem not at all important	○	○	○	○	○	essential

Does your paper address subitem 5-vii? *

Copy and paste relevant sections from the manuscript (include quotes in quotation marks "like this" to indicate direct quotes from your manuscript), or elaborate on this item by providing additional information not in the ms, or briefly explain why the item is not applicable/relevant for your study

Participants accessed the stimuli via Qualtrics.

5-viii) Mode of delivery, features/functionalities/components of the intervention and comparator, and the theoretical framework

Describe mode of delivery, features/functionalities/components of the intervention and comparator, and the theoretical framework [6] used to design them (instructional strategy [1], behaviour change techniques, persuasive features, etc., see e.g., [7, 8] for terminology). This includes an in-depth description of the content (including where it is coming from and who developed it) [1],” whether [and how] it is tailored to individual circumstances and allows users to track their progress and receive feedback” [6]. This also includes a description of communication delivery channels and – if computer-mediated communication is a component – whether communication was synchronous or asynchronous [6]. It also includes information on presentation strategies [1], including page design principles, average amount of text on pages, presence of hyperlinks to other resources, etc. [1].

	1	2	3	4	5	
subitem not at all important	○	○	○	○	○	essential

Does your paper address subitem 5-viii? *

Copy and paste relevant sections from the manuscript (include quotes in quotation marks "like this" to indicate direct quotes from your manuscript), or elaborate on this item by providing additional information not in the ms, or briefly explain why the item is not applicable/relevant for your study

"Following consent, participants were presented a cover story to read. On the basis of the type of physician that they were assigned to, participants were asked to imagine themselves in a situation looking for either a primary care physician or a surgeon. The vignette about a primary care physician depicted a situation that the participant recently moved to a new city and was in need of a new primary care physician. Owing to a lack of input from family members and friends, they decided to search for primary care physicians on PRWs. The vignette about a surgeon described a situation in which the participant had lasting back pains. The primary care physician suspected that the patient may need spinal surgery and provided a list of surgeons to choose from. The participant decided to search for the recommended surgeons on PRWs. After reading through the scenario and imaging themselves in the described situation, each participant was directed to a physician review page to learn about the physician.
A total of 16 physician review pages were developed for this study (see Figure 1). The top part of each page listed basic information about a physician, including the physician’s name (Dr J Smith), the specialty (family medicine or surgeon), and information on new patient acceptance (accepting new patients). To manipulate the type of a physician, half of the Web pages listed the physician’s specialty as family medicine and the other half described the physician as a surgeon.
Each page contained 4 aggregated rating categories about Dr Smith, including 2 items on technical skills (“My doctor accurately diagnosed my problem” and “My doctor effectively treated my problem”) and 2 on interpersonal skills (“My doctor was caring” and “My doctor spent enough time with me”). Past research has suggested that a physician’s skills on diagnosis and treatment are among the most important considerations when selecting a physician [32]. In addition, a physician’s personal manner as well as time spent with a patient are critical to a patient’s satisfaction on the physician’s interpersonal skills [32,33]. These 4 categories frequently appear on PRWs [34] and thus are adopted in this study. To manipulate the valence of physician ratings, these rating categories were assigned different star ratings. Each rating category was presented in the form of aggregated ratings. In the conditions where a physician received high ratings on technical skills, the 2 items pertaining to technical skills were given 5/5-star ratings. In the conditions of moderate ratings on technical skills, the same items were assigned 3/5-star ratings. We chose to examine moderate ratings instead of low ratings in this study because research suggests that low ratings are relatively uncommon on PRWs . The valence of a physician’s interpersonal skills was manipulated in the same way. Furthermore, [illegible]

the rating categories were presented to participants in counterbalanced order to control for the impact of rating order effects. In half of the experimental conditions, the 2 rating categories on technical skills were displayed before the 2 categories on interpersonal skills. In the other half, ratings on technical skills were presented beneath the ratings on interpersonal skills. "

5-ix) Describe use parameters

Describe use parameters (e.g., intended “doses” and optimal timing for use). Clarify what instructions or recommendations were given to the user, e.g., regarding timing, frequency, heaviness of use, if any, or was the intervention used ad libitum.

	1	2	3	4	5	
subitem not at all important	○	○	○	○	○	essential

Does your paper address subitem 5-ix?

Copy and paste relevant sections from the manuscript (include quotes in quotation marks "like this" to indicate direct quotes from your manuscript), or elaborate on this item by providing additional information not in the ms, or briefly explain why the item is not applicable/relevant for your study

Participants were instructed to carefully read the stimuli before completing the survey.

5-x) Clarify the level of human involvement

Clarify the level of human involvement (care providers or health professionals, also technical assistance) in the e-intervention or as co-intervention (detail number and expertise of professionals involved, if any, as well as “type of assistance offered, the timing and frequency of the support, how it is initiated, and the medium by which the assistance is delivered”. It may be necessary to distinguish between the level of human involvement required for the trial, and the level of human involvement required for a routine application outside of a RCT setting (discuss under item 21 – generalizability).

	1	2	3	4	5	
subitem not at all important	○	○	○	○	○	essential

Does your paper address subitem 5-x?

Copy and paste relevant sections from the manuscript (include quotes in quotation marks "like this" to indicate direct quotes from your manuscript), or elaborate on this item by providing additional information not in the ms, or briefly explain why the item is not applicable/relevant for your study

fully-automated.

5-xi) Report any prompts/reminders used

Report any prompts/reminders used: Clarify if there were prompts (letters, emails, phone calls, SMS) to use the application, what triggered them, frequency etc. It may be necessary to distinguish between the level of prompts/reminders required for the trial, and the level of prompts/reminders for a routine application outside of a RCT setting (discuss under item 21 – generalizability).

	1	2	3	4	5	
subitem not at all important	○	○	○	○	○	essential

Does your paper address subitem 5-xi? *

Copy and paste relevant sections from the manuscript (include quotes in quotation marks "like this" to indicate direct quotes from your manuscript), or elaborate on this item by providing additional information not in the ms, or briefly explain why the item is not applicable/relevant for your study

Not used.

5-xii) Describe any co-interventions (incl. training/support)

Describe any co-interventions (incl. training/support): Clearly state any interventions that are provided in addition to the targeted eHealth intervention, as ehealth intervention may not be designed as stand-alone intervention. This includes training sessions and support [1]. It may be necessary to distinguish between the level of training required for the trial, and the level of training for a routine application outside of a RCT setting (discuss under item 21 – generalizability.

	1	2	3	4	5	
subitem not at all important	○	○	○	○	○	essential

Does your paper address subitem 5-xii? *

Copy and paste relevant sections from the manuscript (include quotes in quotation marks "like this" to indicate direct quotes from your manuscript), or elaborate on this item by providing additional information not in the ms, or briefly explain why the item is not applicable/relevant for your study

No co-interventions

6a) Completely defined pre-specified primary and secondary outcome measures, including how and when they were assessed

Does your paper address CONSORT subitem 6a? *

Copy and paste relevant sections from the manuscript (include quotes in quotation marks "like this" to indicate direct quotes from your manuscript), or elaborate on this item by providing additional information not in the ms, or briefly explain why the item is not applicable/relevant for your study

"Willingness to Choose a Physician
A participant’s intention to choose the reviewed physician was assessed with 3 items on a 7-point scale (1=would definitely not choose/definitely unwilling; 7=would definitely choose/definitely willing). The 3 items are “How likely is it that you would choose someone like Dr Smith to be your primary care doctor or surgeon?” (depending on the assigned physician condition), “How willing would you be to go to a doctor like Dr Smith for your medical care?”, and “How willing would you be to recommend a doctor like Dr Smith to your family member and friends if they have the need?” An exploratory factor analysis yielded only 1 factor with an eigenvalue greater than 1, explaining 94.17% of the total variance. All 3 items have factor loadings above .90. The items were then averaged to create a composite variable (mean 4.23, SD 1.79, alpha=.97).
Manipulation Checks
Perceptions of a Physician’s Technical Skills
To determine whether or not the manipulation of a physician’s technical skills was successful, 7 items were used to assess participants’ perceptions of this aspect (eg, knowledgeable, competent, and skilled). Participants were asked how well each of the 7 items described Dr Smith on a 7-point scale (1=very poorly, 7=very well). The items were averaged (mean 5.42, SD 1.34, alpha=.98). As predicted, participants assigned to conditions of high technical skills (mean 6.39, SD 0.75) perceived the physician to be more skilled technically compared with those assigned to conditions of moderate technical skills (mean 4.44, SD 1.06, t483.47=24.78, P<.001).
Perceptions of a Physician’s Interpersonal Skills
Another set of 7 items was used to measure participants’ perceptions of Dr Smith’s interpersonal skills (eg, Kind, Friendly, and Easy to talk to) on a 7-point scale (1=very poorly, 7=very well; mean 5.26, SD 1.24, alpha=.96). As predicted, participants assigned to conditions of high interpersonal skills (mean 6.15, SD 0.79) perceived physicians to be more skilled interpersonally compared with those assigned to conditions of moderate interpersonal skills (mean 4.38, SD 0.95, t520.51=23.54, P<.001).
Control Variables
Current search for a physician. Participants were asked 2 questions to determine whether they were currently searching or recently intending to search for a primary care physician or back surgeon, dependent on the condition they were assigned to (eg, “How likely are you to try and find a new primary care physician or back surgeon in the next twelve months?”). They answered the questions on a 7-point scale (1=will [illegible]

definitely not; 7=will definitely; mean 2.88, SD 1.59).
Past Experience With a Physician
Participants were also asked 2 questions about their past experiences about looking for or having a primary care physician or back surgeon based on the physician type they were assigned to (eg, “Have you ever had a primary care physician or back surgeon?”, “Have you ever searched for a primary care physician or back surgeon?”). Participants answered either yes (1) or no (2) to both questions. For participants assigned to conditions involving a primary care physician, 76.6% (209/273) reported that they have had a primary care physician and 76.2% (208/273) reported that they have searched for a primary care physician. For participants assigned to conditions involving a back surgeon, only 5.2% (14/267) reported that they have had a back surgeon and 12.4% (33/267) reported that they have searched for a back surgeon.
Perceived Reliability of Ratings
Previous research has suggested that people may perceive the reliability of Web-based ratings differently [3,35], which, in turn, may affect their willingness to choose a physician. To control for the variation, participants were asked 1 question to assess the extent to which they consider the Web-based ratings reliable (ie, “To what extent do you consider the patient ratings are reliable measures of Dr Smith’s quality?”). The item was rated on a 7-point scale with the anchors 1=not reliable at all and 7=completely reliable (mean 5.14, SD 1.05).
"

6a-i) Online questionnaires: describe if they were validated for online use and apply CHERRIES items to describe how the questionnaires were designed/deployed

If outcomes were obtained through online questionnaires, describe if they were validated for online use and apply CHERRIES items to describe how the questionnaires were designed/deployed [9].

	1	2	3	4	5	
subitem not at all important	○	○	○	○	○	essential

Does your paper address subitem 6a-i?

Copy and paste relevant sections from manuscript text

"Willingness to Choose a Physician
A participant's intention to choose the reviewed physician was assessed with 3 items on a 7-point scale (1=would definitely not choose/definitely unwilling; 7=would definitely choose/definitely willing). The 3 items are "How likely is it that you would choose someone like Dr Smith to be your primary care doctor or surgeon?" (depending on the assigned physician condition), "How willing would you be to go to a doctor like Dr Smith for your medical care?", and "How willing would you be to recommend a doctor like Dr Smith to your family member and friends if they have the need?" An exploratory factor analysis yielded only 1 factor with an eigenvalue greater than 1, explaining 94.17% of the total variance. All 3 items have factor loadings above .90. The items were then averaged to create a composite variable (mean 4.23, SD 1.79, alpha=.97).
Manipulation Checks
Perceptions of a Physician's Technical Skills
To determine whether or not the manipulation of a physician's technical skills was successful, 7 items were used to assess participants' perceptions of this aspect (eg, knowledgeable, competent, and skilled). Participants were asked how well each of the 7 items described Dr Smith on a 7-point scale (1=very poorly, 7=very well). The items were averaged (mean 5.42, SD 1.34, alpha=.98). As predicted, participants assigned to conditions of high technical skills (mean 6.39, SD 0.75) perceived the physician to be more skilled technically compared with those assigned to conditions of moderate technical skills (mean 4.44, SD 1.06, t483.47=24.78, P<.001).
Perceptions of a Physician's Interpersonal Skills
Another set of 7 items was used to measure participants' perceptions of Dr Smith's interpersonal skills (eg, Kind, Friendly, and Easy to talk to) on a 7-point scale (1=very poorly, 7=very well; mean 5.26, SD 1.24, alpha=.96). As predicted, participants assigned to conditions of high interpersonal skills (mean 6.15, SD 0.79) perceived physicians to be more skilled interpersonally compared with those assigned to conditions of moderate interpersonal skills (mean 4.38, SD 0.95, t520.51=23.54, P<.001).
Control Variables
Current search for a physician. Participants were asked 2 questions to determine whether they were currently searching or recently intending to search for a primary care physician or back surgeon, dependent on the condition they were assigned to (eg, "How likely are you to try and find a new primary care physician or back surgeon in the next twelve months?"). They answered the questions on a 7-point scale (1=will definitely not; 7=will definitely; mean 2.88, SD 1.59).
Past Experience With a Physician

Participants were also asked 2 questions about their past experiences about looking for or having a primary care physician or back surgeon based on the physician type they were assigned to (eg, “Have you ever had a primary care physician or back surgeon?”, “Have you ever searched for a primary care physician or back surgeon?”). Participants answered either yes (1) or no (2) to both questions. For participants assigned to conditions involving a primary care physician, 76.6% (209/273) reported that they have had a primary care physician and 76.2% (208/273) reported that they have searched for a primary care physician. For participants assigned to conditions involving a back surgeon, only 5.2% (14/267) reported that they have had a back surgeon and 12.4% (33/267) reported that they have searched for a back surgeon.
Perceived Reliability of Ratings
Previous research has suggested that people may perceive the reliability of Web-based ratings differently [3,35], which, in turn, may affect their willingness to choose a physician. To control for the variation, participants were asked 1 question to assess the extent to which they consider the Web-based ratings reliable (ie, “To what extent do you consider the patient ratings are reliable measures of Dr Smith’s quality?”). The item was rated on a 7-point scale with the anchors 1=not reliable at all and 7=completely reliable (mean 5.14, SD 1.05).
"

6a-ii) Describe whether and how “use” (including intensity of use/dosage) was defined/measured/monitored

Describe whether and how “use” (including intensity of use/dosage) was defined/measured/monitored (logins, logfile analysis, etc.). Use/adoption metrics are important process outcomes that should be reported in any ehealth trial.

	1	2	3	4	5	
subitem not at all important	○	○	○	○	○	essential

Does your paper address subitem 6a-ii?

Copy and paste relevant sections from manuscript text

Use means reviewing physician ratings sites.

6a-iii) Describe whether, how, and when qualitative feedback from participants was obtained

Describe whether, how, and when qualitative feedback from participants was obtained (e.g., through emails, feedback forms, interviews, focus groups).

	1	2	3	4	5	
subitem not at all important	○	○	○	○	○	essential

Does your paper address subitem 6a-iii?

Copy and paste relevant sections from manuscript text

No qualitative feedback from participants was obtained.

6b) Any changes to trial outcomes after the trial commenced, with reasons

Does your paper address CONSORT subitem 6b? *

Copy and paste relevant sections from the manuscript (include quotes in quotation marks "like this" to indicate direct quotes from your manuscript), or elaborate on this item by providing additional information not in the ms, or briefly explain why the item is not applicable/relevant for your study

No changes were made.

7a) How sample size was determined

NPT: When applicable, details of whether and how the clustering by care provides or centers was addressed

7a-i) Describe whether and how expected attrition was taken into account when calculating the sample size

Describe whether and how expected attrition was taken into account when calculating the sample size.

	1	2	3	4	5	
subitem not at all important	○	○	○	○	○	essential

Does your paper address subitem 7a-i?

Copy and paste relevant sections from manuscript title (include quotes in quotation marks "like this" to indicate direct quotes from your manuscript), or elaborate on this item by providing additional information not in the ms, or briefly explain why the item is not applicable/relevant for your study

Sample size was determined based on the number of experimental conditions.

7b) When applicable, explanation of any interim analyses and stopping guidelines

Does your paper address CONSORT subitem 7b? *

Copy and paste relevant sections from the manuscript (include quotes in quotation marks "like this" to indicate direct quotes from your manuscript), or elaborate on this item by providing additional information not in the ms, or briefly explain why the item is not applicable/relevant for your study

Not applicable.

8a) Method used to generate the random allocation sequence

NPT: When applicable, how care providers were allocated to each trial group

Does your paper address CONSORT subitem 8a? *

Copy and paste relevant sections from the manuscript (include quotes in quotation marks "like this" to indicate direct quotes from your manuscript), or elaborate on this item by providing additional information not in the ms, or briefly explain why the item is not applicable/relevant for your study

Randomiastion function in Qualtrics was used.

8b) Type of randomisation; details of any restriction (such as blocking and block size)

Does your paper address CONSORT subitem 8b? *

Copy and paste relevant sections from the manuscript (include quotes in quotation marks "like this" to indicate direct quotes from your manuscript), or elaborate on this item by providing additional information not in the ms, or briefly explain why the item is not applicable/relevant for your study

participants were randomly assigned to one experimental condition. Most survey items were randomized.

9) Mechanism used to implement the random allocation sequence (such as sequentially numbered containers), describing any steps taken to conceal the sequence until interventions were assigned

Does your paper address CONSORT subitem 9? *

Copy and paste relevant sections from the manuscript (include quotes in quotation marks "like this" to indicate direct quotes from your manuscript), or elaborate on this item by providing additional information not in the ms, or briefly explain why the item is not applicable/relevant for your study

Not applicable

10) Who generated the random allocation sequence, who enrolled participants, and who assigned participants to interventions

Does your paper address CONSORT subitem 10? *

Copy and paste relevant sections from the manuscript (include quotes in quotation marks "like this" to indicate direct quotes from your manuscript), or elaborate on this item by providing additional information not in the ms, or briefly explain why the item is not applicable/relevant for your study

The survey system Qualtrics did the above tasks.

11a) If done, who was blinded after assignment to interventions (for example, participants, care providers, those assessing outcomes) and how

NPT: Whether or not administering co-interventions were blinded to group assignment

11a-i) Specify who was blinded, and who wasn't

Specify who was blinded, and who wasn't. Usually, in web-based trials it is not possible to blind the participants [1, 3] (this should be clearly acknowledged), but it may be possible to blind outcome assessors, those doing data analysis or those administering co-interventions (if any).

	1	2	3	4	5	
subitem not at all important	○	○	○	○	○	essential

Does your paper address subitem 11a-i? *

Copy and paste relevant sections from the manuscript (include quotes in quotation marks "like this" to indicate direct quotes from your manuscript), or elaborate on this item by providing additional information not in the ms, or briefly explain why the item is not applicable/relevant for your study

participants

11a-ii) Discuss e.g., whether participants knew which intervention was the "intervention of interest" and which one was the "comparator"

Informed consent procedures (4a-ii) can create biases and certain expectations - discuss e.g., whether participants knew which intervention was the "intervention of interest" and which one was the "comparator".

	1	2	3	4	5	
subitem not at all important	○	○	○	○	○	essential

Does your paper address subitem 11a-ii?

Copy and paste relevant sections from the manuscript (include quotes in quotation marks "like this" to indicate direct quotes from your manuscript), or elaborate on this item by providing additional information not in the ms, or briefly explain why the item is not applicable/relevant for your study

No, they didn't know the differences.

11b) If relevant, description of the similarity of interventions

(this item is usually not relevant for ehealth trials as it refers to similarity of a placebo or sham intervention to a active medication/intervention)

Does your paper address CONSORT subitem 11b? *

Copy and paste relevant sections from the manuscript (include quotes in quotation marks "like this" to indicate direct quotes from your manuscript), or elaborate on this item by providing additional information not in the ms, or briefly explain why the item is not applicable/relevant for your study

not applicable

12a) Statistical methods used to compare groups for primary and secondary outcomes

NPT: When applicable, details of whether and how the clustering by care providers or centers was addressed

Does your paper address CONSORT subitem 12a? *

Copy and paste relevant sections from the manuscript (include quotes in quotation marks "like this" to indicate direct quotes from your manuscript), or elaborate on this item by providing additional information not in the ms, or briefly explain why the item is not applicable/relevant for your study

"We conducted a 4-way (valence of technical skills × valence of interpersonal skills × physician type × orders of ratings) analysis of covariance (ANCOVA) on people's willingness to choose a physician, controlling for current searching for a physician, past experience with a physician, and perceived reliability of Web-based ratings. As the order of ratings did not affect people's willingness to choose a physician, $F_{1,521}$=.017, P=.90, this factor was not examined further in subsequent analyses. After conducting the ANCOVA, a planned comparison t test was conducted to examine the research question on whether people place more weight on technical or interpersonal skills when selecting a physician. H1 and H2 were tested with tests of simple main effects. All analyses were run using SPSS Statistics version 25. The significance level to reject a null hypothesis was set to .05 for all analyses."

12a-i) Imputation techniques to deal with attrition / missing values

Imputation techniques to deal with attrition / missing values: Not all participants will use the intervention/comparator as intended and attrition is typically high in ehealth trials. Specify how participants who did not use the application or dropped out from the trial were treated in the statistical analysis (a complete case analysis is strongly discouraged, and simple imputation techniques such as LOCF may also be problematic [4]).

	1	2	3	4	5	
subitem not at all important	○	○	○	○	○	essential

Does your paper address subitem 12a-i? *

Copy and paste relevant sections from the manuscript (include quotes in quotation marks "like this" to indicate direct quotes from your manuscript), or elaborate on this item by providing additional information not in the ms, or briefly explain why the item is not applicable/relevant for your study

Not applicable

12b) Methods for additional analyses, such as subgroup analyses and adjusted analyses

Does your paper address CONSORT subitem 12b? *

Copy and paste relevant sections from the manuscript (include quotes in quotation marks "like this" to indicate direct quotes from your manuscript), or elaborate on this item by providing additional information not in the ms, or briefly explain why the item is not applicable/relevant for your study

Not applicable.

X26) REB/IRB Approval and Ethical Considerations [recommended as subheading under "Methods"] (not a CONSORT item)

X26-i) Comment on ethics committee approval

	1	2	3	4	5	
subitem not at all important	○	○	○	○	○	essential

Does your paper address subitem X26-i?

Copy and paste relevant sections from the manuscript (include quotes in quotation marks "like this" to indicate direct quotes from your manuscript), or elaborate on this item by providing additional information not in the ms, or briefly explain why the item is not applicable/relevant for your study

The Institutional Review Board at the Ohio State University approved all study procedures.

x26-ii) Outline informed consent procedures

Outline informed consent procedures e.g., if consent was obtained offline or online (how? Checkbox, etc.?), and what information was provided (see 4a-ii). See [6] for some items to be included in informed consent documents.

	1	2	3	4	5	
subitem not at all important	○	○	○	○	○	essential

Does your paper address subitem X26-ii?

Copy and paste relevant sections from the manuscript (include quotes in quotation marks "like this" to indicate direct quotes from your manuscript), or elaborate on this item by providing additional information not in the ms, or briefly explain why the item is not applicable/relevant for your study

Consent was obtained online.

X26-iii) Safety and security procedures

Safety and security procedures, incl. privacy considerations, and any steps taken to reduce the likelihood or detection of harm (e.g., education and training, availability of a hotline)

	1	2	3	4	5	
subitem not at all important	○	○	○	○	○	essential

Does your paper address subitem X26-iii?

Copy and paste relevant sections from the manuscript (include quotes in quotation marks "like this" to indicate direct quotes from your manuscript), or elaborate on this item by providing additional information not in the ms, or briefly explain why the item is not applicable/relevant for your study

Data were collected anonymously.

RESULTS

13a) For each group, the numbers of participants who were randomly assigned, received intended treatment, and were analysed for the primary outcome

NPT: The number of care providers or centers performing the intervention in each group and the number of patients treated by each care provider in each center

Does your paper address CONSORT subitem 13a? *

Copy and paste relevant sections from the manuscript (include quotes in quotation marks "like this" to indicate direct quotes from your manuscript), or elaborate on this item by providing additional information not in the ms, or briefly explain why the item is not applicable/relevant for your study

The information was provided in Table 1.

13b) For each group, losses and exclusions after randomisation, together with reasons

Does your paper address CONSORT subitem 13b? (NOTE: Preferably, this is shown in a CONSORT flow diagram) *

Copy and paste relevant sections from the manuscript (include quotes in quotation marks "like this" to indicate direct quotes from your manuscript), or elaborate on this item by providing additional information not in the ms, or briefly explain why the item is not applicable/relevant for your study

"A total of 608 participants completed the Web-based experiment. Participants were recruited via the crowdsourcing website, Amazon's Mechanical Turk (mTurk), and compensated for their time. We excluded people who failed the attention checks (n=26) and those who spent no time (n=1) or less than 5 seconds on the Web page (n=41). "

13b-i) Attrition diagram

Strongly recommended: An attrition diagram (e.g., proportion of participants still logging in or using the intervention/comparator in each group plotted over time, similar to a survival curve) or other figures or tables demonstrating usage/dose/engagement.

	1	2	3	4	5	
subitem not at all important	○	○	○	○	○	essential

Does your paper address subitem 13b-i?

Copy and paste relevant sections from the manuscript or cite the figure number if applicable (include quotes in quotation marks "like this" to indicate direct quotes from your manuscript), or elaborate on this item by providing additional information not in the ms, or briefly explain why the item is not applicable/relevant for your study

Your answer

14a) Dates defining the periods of recruitment and follow-up

Does your paper address CONSORT subitem 14a? *

Copy and paste relevant sections from the manuscript (include quotes in quotation marks "like this" to indicate direct quotes from your manuscript), or elaborate on this item by providing additional information not in the ms, or briefly explain why the item is not applicable/relevant for your study

March - May 2017

14a-i) Indicate if critical “secular events” fell into the study period

Indicate if critical “secular events” fell into the study period, e.g., significant changes in Internet resources available or “changes in computer hardware or Internet delivery resources”

	1	2	3	4	5	
subitem not at all important	○	○	○	○	○	essential

Does your paper address subitem 14a-i?

Copy and paste relevant sections from the manuscript (include quotes in quotation marks "like this" to indicate direct quotes from your manuscript), or elaborate on this item by providing additional information not in the ms, or briefly explain why the item is not applicable/relevant for your study

Your answer

14b) Why the trial ended or was stopped (early)

Does your paper address CONSORT subitem 14b? *

Copy and paste relevant sections from the manuscript (include quotes in quotation marks "like this" to indicate direct quotes from your manuscript), or elaborate on this item by providing additional information not in the ms, or briefly explain why the item is not applicable/relevant for your study

No.

15) A table showing baseline demographic and clinical characteristics for each group

NPT: When applicable, a description of care providers (case volume, qualification, expertise, etc.) and centers (volume) in each group

Does your paper address CONSORT subitem 15? *

Copy and paste relevant sections from the manuscript (include quotes in quotation marks "like this" to indicate direct quotes from your manuscript), or elaborate on this item by providing additional information not in the ms, or briefly explain why the item is not applicable/relevant for your study

The information was provided in Table 1.

15-i) Report demographics associated with digital divide issues

In ehealth trials it is particularly important to report demographics associated with digital divide issues, such as age, education, gender, social-economic status, computer/Internet/ehealth literacy of the participants, if known.

	1	2	3	4	5	
subitem not at all important	○	○	○	○	○	essential

Does your paper address subitem 15-i? *

Copy and paste relevant sections from the manuscript (include quotes in quotation marks "like this" to indicate direct quotes from your manuscript), or elaborate on this item by providing additional information not in the ms, or briefly explain why the item is not applicable/relevant for your study

The information was provided in Table 1.

16) For each group, number of participants (denominator) included in each analysis and whether the analysis was by original assigned groups

16-i) Report multiple “denominators” and provide definitions

Report multiple “denominators” and provide definitions: Report N’s (and effect sizes) “across a range of study participation [and use] thresholds” [1], e.g., N exposed, N consented, N used more than x times, N used more than y weeks, N participants “used” the intervention/comparator at specific pre-defined time points of interest (in absolute and relative numbers per group). Always clearly define “use” of the intervention.

	1	2	3	4	5	
subitem not at all important	○	○	○	○	○	essential

Does your paper address subitem 16-i? *

Copy and paste relevant sections from the manuscript (include quotes in quotation marks "like this" to indicate direct quotes from your manuscript), or elaborate on this item by providing additional information not in the ms, or briefly explain why the item is not applicable/relevant for your study

The denominators were reported in the manuscript.

16-ii) Primary analysis should be intent-to-treat

Primary analysis should be intent-to-treat, secondary analyses could include comparing only “users”, with the appropriate caveats that this is no longer a randomized sample (see 18-i).

	1	2	3	4	5	
subitem not at all important	○	○	○	○	○	essential

Does your paper address subitem 16-ii?

Copy and paste relevant sections from the manuscript (include quotes in quotation marks "like this" to indicate direct quotes from your manuscript), or elaborate on this item by providing additional information not in the ms, or briefly explain why the item is not applicable/relevant for your study

Your answer

17a) For each primary and secondary outcome, results for each group, and the estimated effect size and its precision (such as 95% confidence interval)

Does your paper address CONSORT subitem 17a? *

Copy and paste relevant sections from the manuscript (include quotes in quotation marks "like this" to indicate direct quotes from your manuscript), or elaborate on this item by providing additional information not in the ms, or briefly explain why the item is not applicable/relevant for your study

"The research question concerns people's willingness to choose a physician with higher ratings on one aspect than the other. The ANCOVA test suggests that the 2-way interaction between ratings of technical skills and ratings of interpersonal skills significantly affected people's willingness to choose a physician, F1,521=30.42, P<.001, ηp2=.06. A planned comparison t test was then conducted to further examine whether people are more willing to choose a physician with higher ratings on technical skills or interpersonal skills. The condition of high ratings on both skills was assigned a weight of 2; the condition of moderate ratings on both skills was assigned a weight of −2; the condition of high ratings on technical skills and moderate ratings on interpersonal skills was assigned a weight of 1; the condition of high ratings on interpersonal skills and moderate ratings on technical skills was assigned a weight of −1. The results suggested that people were significantly more likely to choose a physician with higher ratings on technical skills than on interpersonal skills (mean 4.79, SD 1.28) compared with a physician with higher ratings on interpersonal skills than on technical skills (mean 3.06, SD 1.55, t369.96=22.36, P<.001, Cohen d=1.22).

The first hypothesis predicted that people had higher intention to choose a specialist who has higher ratings on technical skills than on interpersonal skills, compared with a primary care physician with the same ratings. A 3-way interaction among ratings of technical skills, ratings of interpersonal skills, and physician type was not significant, F1,521=3.68, P=.06, ηp2=.01. A posthoc analysis was then conducted to test the simple main effects of physician types within the interaction of technical and interpersonal skills. As predicted, participants were more willing to choose a specialist with higher ratings on technical skills than on interpersonal skills (mean 5.07, SD 1.38) compared with a primary care physician with the same ratings (mean 4.50, SD 1.36), F1,521=5.34, P=.02. Hence, H1 was supported.

The second hypothesis proposed that people were more willing to choose a primary care physician who has higher ratings on interpersonal skills than on technical skills, compared with a specialist with the same ratings. Contradictory to the prediction, the test of simple main effects suggested that people did not differ in their willingness to select a primary care physician (mean 3.09, SD 1.40) and a specialist (mean 3.06, SD 1.39) when the physician had higher ratings on interpersonal skills than on technical skills, F1, 521=0.013, P=.91. Therefore, H2 was not supported."

17a-i) Presentation of process outcomes such as metrics of use and intensity of use

In addition to primary/secondary (clinical) outcomes, the presentation of process outcomes such as metrics of use and intensity of use (dose, exposure) and their operational definitions is critical. This does not only refer to metrics of attrition (13-b) (often a binary variable), but also to more continuous exposure metrics such as "average session length". These must be accompanied by a technical description how a metric like a "session" is defined (e.g., timeout after idle time) [1] (report under item 6a).

	1	2	3	4	5	
subitem not at all important	○	○	○	○	○	essential

Does your paper address subitem 17a-i?

Copy and paste relevant sections from the manuscript (include quotes in quotation marks "like this" to indicate direct quotes from your manuscript), or elaborate on this item by providing additional information not in the ms, or briefly explain why the item is not applicable/relevant for your study

Your answer

17b) For binary outcomes, presentation of both absolute and relative effect sizes is recommended

Does your paper address CONSORT subitem 17b? *

Copy and paste relevant sections from the manuscript (include quotes in quotation marks "like this" to indicate direct quotes from your manuscript), or elaborate on this item by providing additional information not in the ms, or briefly explain why the item is not applicable/relevant for your study

Not applicable.

18) Results of any other analyses performed, including subgroup analyses and adjusted analyses, distinguishing pre-specified from exploratory

Does your paper address CONSORT subitem 18? *

Copy and paste relevant sections from the manuscript (include quotes in quotation marks "like this" to indicate direct quotes from your manuscript), or elaborate on this item by providing additional information not in the ms, or briefly explain why the item is not applicable/relevant for your study

Data were provided in the results section.

18-i) Subgroup analysis of comparing only users

A subgroup analysis of comparing only users is not uncommon in ehealth trials, but if done, it must be stressed that this is a self-selected sample and no longer an unbiased sample from a randomized trial (see 16-iii).

	1	2	3	4	5	
subitem not at all important	○	○	○	○	○	essential

Does your paper address subitem 18-i?

Copy and paste relevant sections from the manuscript (include quotes in quotation marks "like this" to indicate direct quotes from your manuscript), or elaborate on this item by providing additional information not in the ms, or briefly explain why the item is not applicable/relevant for your study

Your answer

19) All important harms or unintended effects in each group

(for specific guidance see CONSORT for harms)

Does your paper address CONSORT subitem 19? *

Copy and paste relevant sections from the manuscript (include quotes in quotation marks "like this" to indicate direct quotes from your manuscript), or elaborate on this item by providing additional information not in the ms, or briefly explain why the item is not applicable/relevant for your study

No important harms or unintended effects in each group.

19-i) Include privacy breaches, technical problems

Include privacy breaches, technical problems. This does not only include physical "harm" to participants, but also incidents such as perceived or real privacy breaches [1], technical problems, and other unexpected/unintended incidents. "Unintended effects" also includes unintended positive effects [2].

	1	2	3	4	5	
subitem not at all important	○	○	○	○	○	essential

Does your paper address subitem 19-i?

Copy and paste relevant sections from the manuscript (include quotes in quotation marks "like this" to indicate direct quotes from your manuscript), or elaborate on this item by providing additional information not in the ms, or briefly explain why the item is not applicable/relevant for your study

Your answer

19-ii) Include qualitative feedback from participants or observations from staff/researchers

Include qualitative feedback from participants or observations from staff/researchers, if available, on strengths and shortcomings of the application, especially if they point to unintended/unexpected effects or uses. This includes (if available) reasons for why people did or did not use the application as intended by the developers.

	1	2	3	4	5	
subitem not at all important	○	○	○	○	○	essential

Does your paper address subitem 19-ii?

Copy and paste relevant sections from the manuscript (include quotes in quotation marks "like this" to indicate direct quotes from your manuscript), or elaborate on this item by providing additional information not in the ms, or briefly explain why the item is not applicable/relevant for your study

Your answer

DISCUSSION

22) Interpretation consistent with results, balancing benefits and harms, and considering other relevant evidence

NPT: In addition, take into account the choice of the comparator, lack of or partial blinding, and unequal expertise of care providers or centers in each group

22-i) Restate study questions and summarize the answers suggested by the data, starting with primary outcomes and process outcomes (use)

Restate study questions and summarize the answers suggested by the data, starting with primary outcomes and process outcomes (use).

	1	2	3	4	5	
subitem not at all important	○	○	○	○	○	essential

Does your paper address subitem 22-i? *

Copy and paste relevant sections from the manuscript (include quotes in quotation marks "like this" to indicate direct quotes from your manuscript), or elaborate on this item by providing additional information not in the ms, or briefly explain why the item is not applicable/relevant for your study

"Patients are increasingly empowered in this rapidly changing health care landscape. With the access to physician reviews on the Web, patients are taking a more active role in their selection of physicians. Physicians and patients have different attitudes toward reviews provided on PRWs [6]. Physicians tend to question the accuracy of Web-based reviews and view them as a threat to their reputations [4], whereas patients generally have a favorable attitude and would consult these reviews in their choice of physicians [3]. It is thus imperative to understand how Web-based reviews affect patients' perceptions and choice of physicians, which may help patients and health professionals have a better understanding of the role of PRWs in health consumerism.

Specifically, this study took the initiative to examine if Web-based physician ratings affect patients' selection of primary care physicians and specialists differently. We investigated how Web-based reviews focusing on physicians' technical and interpersonal skills affect people's intention to select different types of physicians. The results showed that people were more willing to choose a physician with higher ratings on technical skills than on interpersonal skills compared with a physician with higher ratings on interpersonal skills than on technical skills. Furthermore, people perceived technical skills as more important and were more willing to compromise on interpersonal skills in their choice of a specialist compared with a primary care physician.

This study contributes to previous research on physician selection via PRWs by experimentally testing one's preference for a physician who is high on technical skills versus interpersonal skills. Apart from previous research that relied on survey measures to assess patient's preference for a physician's skills, little work has experimentally tested the preference. By presenting patients with a mockup physician review site and a medical care vignette, we are able to aid the patients in imagining themselves in a medical situation and thus make their preferences more accessible. Before this study, it was unclear how specific factors such as rating categories or physician characteristics may affect people's choice of physicians on PRWs [14]. With an experimental design, physician types and rating categories could be separately operationalized and directly compared to examine their role in people's choice of physicians. "

22-ii) Highlight unanswered new questions, suggest future research

Highlight unanswered new questions, suggest future research.

	1	2	3	4	5	
subitem not at all important	○	○	○	○	○	essential

Does your paper address subitem 22-ii?

Copy and paste relevant sections from the manuscript (include quotes in quotation marks "like this" to indicate direct quotes from your manuscript), or elaborate on this item by providing additional information not in the ms, or briefly explain why the item is not applicable/relevant for your study

Your answer

20) Trial limitations, addressing sources of potential bias, imprecision, and, if relevant, multiplicity of analyses

20-i) Typical limitations in ehealth trials

Typical limitations in ehealth trials: Participants in ehealth trials are rarely blinded. Ehealth trials often look at a multiplicity of outcomes, increasing risk for a Type I error. Discuss biases due to non-use of the intervention/usability issues, biases through informed consent procedures, unexpected events.

	1	2	3	4	5	
subitem not at all important	○	○	○	○	○	essential

Does your paper address subitem 20-i? *

Copy and paste relevant sections from the manuscript (include quotes in quotation marks "like this" to indicate direct quotes from your manuscript), or elaborate on this item by providing additional information not in the ms, or briefly explain why the item is not applicable/relevant for your study

”This study has several limitations that point to directions for future research. First, because previous research suggests that only a small proportion of reviews on PRWs are negative [7,38], this study did not include negative ratings. Although we deliberately excluded negative ratings to represent the reality of PRWs, it would still be worthwhile to learn how negative ratings may affect people’s choice of physicians. In particular, negativity effects may take place such that patients are more impacted by negative ratings than positive ones on their selection of physicians. Under such circumstances, people may not be willing to choose a technically skilled physician who receives negative feedback on interpersonal skills.

Second, this study examined the impact of numerical ratings, but not narrative comments, on patients’ willingness to choose physicians. Although patients’ evaluations are primarily displayed in the format of aggregated numerical ratings on PRWs, many portals also allow patients to leave narrative comments to detail their satisfaction and dissatisfaction. Aggregated numerical ratings tend to provide patients a holistic view of physicians and the services they provide. Narrative comments, on the contrary, can capture more detailed and nuanced feedback that is not reflected in structured rating systems. A direction for future research is to investigate how numerical ratings and narrative comments work together to affect people’s willingness to choose a physician, especially if 2 sources present contradictory information.

Third, this study focused on rating categories pertaining to a physician’s technical and interpersonal skills. In selection of a physician, patients take into account many considerations beyond a physician’s qualifications. For example, previous research has found that management practices such as punctuality and staff quality are also considered in patients’ choice of physicians [31,39]. Besides reviewing a physician’s qualifications, many PRWs also include rating categories on management practices. Future research should look into these aspects in addition to a physician’s skills.

Fourth, despite a wide range of specialties, this study operationalized a specialist to be a back surgeon. However, it is likely that patients use different selection criteria for specialists of different types. Under certain circumstances, patients may place more weightage on a specialist’s interpersonal skills than technical skills (eg, visiting a gynecologist for a check-up). Future research thus needs to examine if the influence of Web-based ratings on physician choice differs as a function of [illegible]

physician specialties and medical conditions.

Finally, a patient’s willingness to choose a physician is influenced by a variety of factors beyond numerical ratings displayed on PRWs. For example, demographic information of a physician (eg, sex and age) and environmental factors (eg, office location) should be taken into account when examining patients’ choice of physicians. Another direction for future research is to explore underlying mechanisms, especially perceptual processes, through which physician types and patient reviews affect people’s choice of physicians. “

21) Generalisability (external validity, applicability) of the trial findings

NPT: External validity of the trial findings according to the intervention, comparators, patients, and care providers or centers involved in the trial

21-i) Generalizability to other populations

Generalizability to other populations: In particular, discuss generalizability to a general Internet population, outside of a RCT setting, and general patient population, including applicability of the study results for other organizations

	1	2	3	4	5	
subitem not at all important	○	○	○	○	○	essential

Does your paper address subitem 21-i?

Copy and paste relevant sections from the manuscript (include quotes in quotation marks "like this" to indicate direct quotes from your manuscript), or elaborate on this item by providing additional information not in the ms, or briefly explain why the item is not applicable/relevant for your study

Your answer

21-ii) Discuss if there were elements in the RCT that would be different in a routine application setting

Discuss if there were elements in the RCT that would be different in a routine application setting (e.g., prompts/reminders, more human involvement, training sessions or other co-interventions) and what impact the omission of these elements could have on use, adoption, or outcomes if the intervention is applied outside of a RCT setting.

	1	2	3	4	5	
subitem not at all important	○	○	○	○	○	essential

Does your paper address subitem 21-ii?

Copy and paste relevant sections from the manuscript (include quotes in quotation marks "like this" to indicate direct quotes from your manuscript), or elaborate on this item by providing additional information not in the ms, or briefly explain why the item is not applicable/relevant for your study

Your answer

OTHER INFORMATION

23) Registration number and name of trial registry

Does your paper address CONSORT subitem 23? *

Copy and paste relevant sections from the manuscript (include quotes in quotation marks "like this" to indicate direct quotes from your manuscript), or elaborate on this item by providing additional information not in the ms, or briefly explain why the item is not applicable/relevant for your study

ISRCTN91316463 "The impact of web-based ratings on physician selection"

24) Where the full trial protocol can be accessed, if available

Does your paper address CONSORT subitem 24? *

Cite a Multimedia Appendix, other reference, or copy and paste relevant sections from the manuscript (include quotes in quotation marks "like this" to indicate direct quotes from your manuscript), or elaborate on this item by providing additional information not in the ms, or briefly explain why the item is not applicable/relevant for your study

It can be accessed upon request to the researchers.

25) Sources of funding and other support (such as supply of drugs), role of funders

Does your paper address CONSORT subitem 25? *

Copy and paste relevant sections from the manuscript (include quotes in quotation marks "like this" to indicate direct quotes from your manuscript), or elaborate on this item by providing additional information not in the ms, or briefly explain why the item is not applicable/relevant for your study

Not applicable.

X27) Conflicts of Interest (not a CONSORT item)

X27-i) State the relation of the study team towards the system being evaluated

In addition to the usual declaration of interests (financial or otherwise), also state the relation of the study team towards the system being evaluated, i.e., state if the authors/evaluators are distinct from or identical with the developers/sponsors of the intervention.

	1	2	3	4	5	
subitem not at all important	○	○	○	○	○	essential

Does your paper address subitem X27-i?

Copy and paste relevant sections from the manuscript (include quotes in quotation marks "like this" to indicate direct quotes from your manuscript), or elaborate on this item by providing additional information not in the ms, or briefly explain why the item is not applicable/relevant for your study

Your answer

About the CONSORT EHEALTH checklist

As a result of using this checklist, did you make changes in your manuscript? *

- ○ yes, major changes
- ◉ yes, minor changes
- ○ no

What were the most important changes you made as a result of using this checklist?

Your answer

How much time did you spend on going through the checklist INCLUDING making changes in your manuscript *

Two hours

As a result of using this checklist, do you think your manuscript has improved? *

- ○ yes
- ○ no
- ◉ Other: Not much. It's time consuming.

Would you like to become involved in the CONSORT EHEALTH group?

This would involve for example becoming involved in participating in a workshop and writing an "Explanation and Elaboration" document

- yes
- no
- Other:

Any other comments or questions on CONSORT EHEALTH

Your answer

STOP - Save this form as PDF before you click submit

To generate a record that you filled in this form, we recommend to generate a PDF of this page (on a Mac, simply select "print" and then select "print as PDF") before you submit it.

When you submit your (revised) paper to JMIR, please upload the PDF as supplementary file.

Don't worry if some text in the textboxes is cut off, as we still have the complete information in our database. Thank you!

Final step: Click submit !

Click submit so we have your answers in our database!

SUBMIT

Never submit passwords through Google Forms.

This content is neither created nor endorsed by Google. Report Abuse - Terms of Service

Google Forms
